# CYP2J2 and its metabolites (epoxyeicosatrienoic acids) attenuate cardiac hypertrophy by activating AMPKα2 and enhancing nuclear translocation of Akt1

**DOI:** 10.1111/acel.12507

**Published:** 2016-07-14

**Authors:** Bei Wang, Hesong Zeng, Zheng Wen, Chen Chen, Dao Wen Wang

**Affiliations:** ^1^ Division of CardiologyDepartment of Internal MedicineTongji HospitalTongji Medical CollegeHuazhong University of Science and TechnologyWuhan430030China

**Keywords:** 11,12‐EET, Akt1, AMPKα2, cardiac hypertrophy, CYP2J2

## Abstract

Cytochrome P450 epoyxgenase 2J2 and epoxyeicosatrienoic acids (EETs) are known to protect against cardiac hypertrophy and heart failure, which involve the activation of 5′‐AMP‐activated protein kinase (AMPK) and Akt. Although the functional roles of AMPK and Akt are well established, the significance of cross talk between them in the development of cardiac hypertrophy and antihypertrophy of CYP2J2 and EETs remains unclear. We investigated whether CYP2J2 and its metabolites EETs protected against cardiac hypertrophy by activating AMPKα2 and Akt1. Moreover, we tested whether EETs enhanced cross talk between AMPKα2 and phosphorylated Akt1 (p‐Akt1), and stimulated nuclear translocation of p‐Akt1, to exert their antihypertrophic effects. AMPKα2^−/−^ mice that overexpressed CYP2J2 in heart were treated with Ang II for 2 weeks. Interestingly, overexpression of CYP2J2 suppressed cardiac hypertrophy and increased levels of atrial natriuretic peptide (ANP) in the heart tissue and plasma of wild‐type mice but not AMPKα2^−/−^ mice. The CYP2J2 metabolites, 11,12‐EET, activated AMPKα2 to induce nuclear translocation of p‐Akt1 selectively, which increased the production of ANP and therefore inhibited the development of cardiac hypertrophy. Furthermore, by co‐immunoprecipitation analysis, we found that AMPKα2β2γ1 and p‐Akt1 interact through the direct binding of the AMPKγ1 subunit to the Akt1 protein kinase domain. This interaction was enhanced by 11,12‐EET. Our studies reveal a novel mechanism in which CYP2J2 and EETs enhanced Akt1 nuclear translocation through interaction with AMPKα2β2γ1 and protect against cardiac hypertrophy and suggest that overexpression of CYP2J2 might have clinical potential to suppress cardiac hypertrophy and heart failure.

## Introduction

The heart responds to enhanced hemodynamic load arising from a variety of physiological and pathophysiological conditions (such as exercise, hypertension, valvular disease, and cardiomyopathy) by undergoing hypertrophy. Cardiac hypertrophy is characterized by an increase in ventricular mass resulting from an increase in cardiomyocyte size (Frey & Olson, 2003). In response to hemodynamic overload, individual cardiomyocytes activate intracellular hypertrophic signaling pathways to reuse embryonic transcription factors and to increase the synthesis of various structural and contractile proteins (Oka *et al*., 2014). Cardiac hypertrophy is believed to be a compensatory or adaptive response of the heart to hemodynamic overload, as it initially reduces cardiac wall stress. However, in pathophysiological conditions, the sustained cardiac ventricular hypertrophy results in increased cardiac stress and leads to an increased risk of heart failure and malignant arrhythmia (Levy *et al*., 1990; Koren *et al*., 1991). Elucidation of the intracellular signaling pathways responsible for these negative outcomes, and of those that protect against such outcomes, may enable the development of new targeted therapies.

Cytochrome P450 (CYP) epoxygenases convert arachidonic acid to four regioisomeric epoxyeicosatrienoic acids (5,6‐EET, 8,9‐EET, 11,12‐EET, and 14,15‐EET), which exert diverse biological activities in the cardiovascular system (Xu *et al*., 2011). CYP epoxygenases and their EET products are known to have vasodilatory, antihypertensive, pro‐angiogenic, anti‐atherosclerotic, and anti‐inflammatory effects and to protect against ischemia–reperfusion injury (Xu *et al*., 2011). There are accumulating evidences that EETs (Althurwi *et al*., 2013; He *et al*., 2015) might also protect against cardiac hypertrophy (Ai *et al*., 2009). Overexpression of CYP epoxygenases improved cardiac function in spontaneously hypertensive rats, and these effects may have been mediated, at least in part, by the atrial natriuretic peptide (ANP) activation of the epidermal growth factor (EGF) receptor (Xiao *et al*., 2010). However, the detailed molecular mechanisms by which EETs protect against cardiac hypertrophy or heart failure remain unclear.

Previous studies have shown that EETs regulate the phosphorylation, and therefore activation, of 5′‐AMP‐activated protein kinase (AMPK) (Xu *et al*., 2010; Samokhvalov *et al*., 2013), a heterotrimeric enzyme with one catalytic (α) and two regulatory (β and γ) subunits. In addition, the activation of AMPK protects against the development of cardiac hypertrophy or cardiac contractile dysfunction through mechanisms including (p‐70S6K^Thr389^)‐(p‐elf4e^Ser209^)‐(p‐4EBP1^Thr46^)‐mediated protein synthesis pathway or associated with AMPK‐mTORC1‐ULK1‐mediated autophagy (Zhang *et al*., 2008; Turdi *et al*., 2011; Guo & Ren, 2012). However, whether EET‐mediated protection against the development of cardiac hypertrophy occurs via the activation of AMPK needs to be further investigated.

Akt/PKB (protein kinase B) is a serine–threonine protein kinase that mediates growth responses and survivals in many cell types (Tuttle *et al*., 2001; Sussman *et al*., 2011). Akt is initially activated at the cell membrane in response to stimulation by growth factors, such as insulin‐like growth factor 1 (IGF1) (Alessi *et al*., 1996). After activation, Akt phosphorylates multiple cytosolic substrates and translocates to nucleus, where it is thought to regulate gene transcription (Andjelkovic *et al*., 1997; Brazil *et al*., 2002). Mammals express three isoforms of Akt; Akt1 and Akt2 are expressed ubiquitously, whereas Akt3 is found predominantly in the brain, kidney, and heart (Datta *et al*., 1999; Masure *et al*., 1999). Several studies have demonstrated a cardioprotective role for Akt1, especially nuclear Akt1, in response to pathological challenges (Sugden & Clerk, 2001; Shiraishi *et al*., 2004; DeBosch *et al*., 2006; Tsujita *et al*., 2006). One study has shown that Akt1‐mediated protection against cardiac hypertrophy is dependent on ANP (Tsujita *et al*., 2006). In this study, we investigated whether the CYP2J2, or EETs, could suppress cardiac hypertrophy by activating AMPKα2.

## Results

### Cardiomyocyte‐specific overexpression of CYP2J2 *in vivo* attenuated myocardial hypertrophy and remodeling

**Figure 1:**
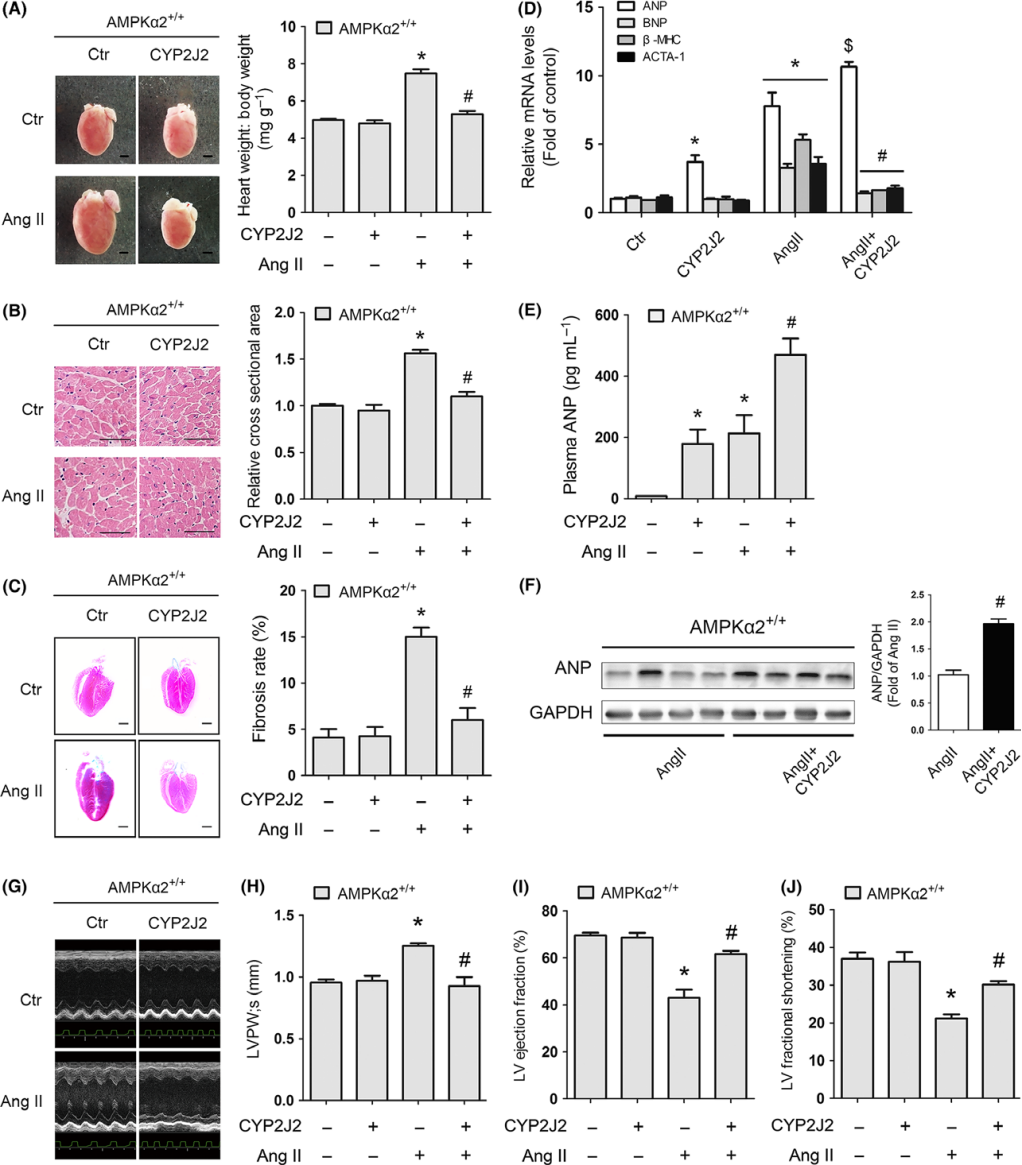
Cardiomyocyte‐specific overexpression of CYP2J2 attenuated myocardial hypertrophy and remodeling *in vivo*. AMPKα2^+/+^ mice were injected in the caudal vein with rAAV9‐CYP2J2. After 2 weeks, mice were exposed to continuous infusion of Ang II or a saline control for 14 days (8–10 mice for each group). (A) Left, gross morphology of adult hearts AMPKα2^+/+^ mice 2 weeks after Ang II infusion. Scale bar: 1 mm. Right, heart weight: body weight ratios of adult WT mice after infusion with Ang II or saline control for 2 weeks. (B) Left, H&E staining of sections of adult hearts from AMPKα2^+/+^ mice after infusion with Ang II or saline control for 2 weeks (Scale bar: 100 μm). Right, quantification of the size of cardiomyocytes by measurement of the cross‐sectional area on H&E‐stained sections. More than 200 cells from four different hearts were analyzed per group. (C) Left, masson trichrome staining of adult hearts from AMPKα2^+/+^ mice after infusion with Ang II or saline control for 2 weeks. The blue area indicates collagen fibers. Scale bar: 1 mm. Right, quantification of the rate of cardiac fibrosis by measurement of the area of collagen deposition. (D) RT–PCR analyses of relative expression of ANP, BNP, β‐MHC, and ACTA1 genes from the hearts of mice exposed to the indicated conditions. (E) ELISA analysis showing the expression of ANP in plasma from AMPKα2^+/+^ mice (*n* = 4–5 for each group). (F) Left, Western blot analyses showing the expression of the ANP protein in Ang II and Ang II+CYP2J2 groups. GAPDH was used as a loading control. Right, the intensity of the Western blot signal was quantified and is shown as relative protein expression after normalization to GAPDH. (G) Representative images of echocardiograms. (H) LVPW;s. (I) LV ejection fraction. (J) LV fractional shortening. The data represent the mean ± SEM from at least four independent experiments (*P < 0.05 vs. control group; ^$^ *P* < 0.05 vs. control group; ^#^ *P* < 0.05 vs. Ang II group).

The recombinant rAAV9 vector was coupled to CYP2J2, and the rAAV9‐CYP2J2 construct was injected into the caudal vein of AMPKα2^+/+^ mice. Western blot analyses performed 1 month after injection showed that rAAV9‐CYP2J2 treatment led to an abundant expression of CYP2J2 in the heart (Fig. S1A) in mice; much lower levels of CYP2J2 expression were observed in liver (Fig. S1B) and skeletal muscle (Fig. S1C), and no expression was detected in kidney (Fig. S1D). Two weeks after injection with rAAV9‐CYP2J2, the mice were exposed to 14 days of continuous infusion of either a saline control or Ang II (1 mg kg^−1^ day^−1^) to induce chronic hypertension and cardiac hypertrophy (Zhong *et al*., 2010). Heart size was evaluated at the end of the 14‐day treatment period. Ang II infusion significantly increased heart size and the heart weight/body weight ratio in AMPKα2^+/+^ mice compared with saline‐treated mice (Fig. 1A,B).

The forced expression of CYP2J2 prevented Ang II‐induced cardiac hypertrophy in mice (Fig. 1A,B). H&E (hematoxylin and eosin) staining of cardiac sections confirmed that Ang II stimulation increased the area of the cardiomyocytes; this effect was prevented by CYP2J2 overexpression in AMPKα2^+/+^ mice (Fig. 1B). Ang II‐induced cardiac fibrosis was also suppressed by overexpression of CYP2J2 (Fig. 1C). Furthermore, CYP2J2 overexpression ameliorated Ang II‐induced expression of the biomarkers of cardiac hypertrophy brain natriuretic peptide (BNP), β‐myosin heavy chain (β‐MHC), and skeletal muscle α‐actin (ACTA1) (Fig. 1D). Ang II treatment led to significantly increased expression of ANP (Figs 1D,E and S4C). Interestingly, CYP2J2 treatment led to higher expression of ANP in mice, regardless of whether they were treated with Ang II (Fig. 1D–F).

Hemodynamic and cardiac functions were also evaluated after 14 days of infusion with Ang II or saline (Figs 1G,F, S2, and Table S3). The heart rates of AMPKα2^+/+^ mice were not affected by Ang II infusion (Fig. S2A and Table S3). Echocardiography showed Ang II induced increases in the thickness of the interventricular septum and left ventricular posterior wall and increases in the left ventricular mass in AMPKα2^+/+^ mice. CYP2J2 overexpression in the heart prevented this hypertrophic response (Figs 1G,H and S2B–C, Table S3). Measurement of left ventricular ejection fraction (Fig. 1I and Table S3) and fractional shortening (Fig. 1J and Table S3) showed that CYP2J2 overexpression prevented Ang II‐induced ventricular systolic dysfunction in mice. Moreover, an Ang II‐induced reduction in cardiac function, demonstrated by decreased *dP*/*dt*_max_ and *dP*/*dt*_min_, was prevented by overexpression of CYP2J2 (Fig. S2D,E).

Together, these results suggest that CYP2J2 overexpression protects against development of cardiac hypertrophy and cardiac remodeling and that CYP2J2‐mediated cardioprotection is accompanied by an increase in ANP expression.

### EETs (especially 11,12‐EET) are responsible for antihypertrophic effect of CYP2J2 overexpression

Significant increases in cardiac 11,12‐EET (Fig. S1E) and 11,12‐DHET (Fig. S1F; representative one of four EETs) levels were observed in AMPKα2^+/+^ mice treated with rAAV9‐CYP2J2. To determine antihypertrophic effects of CYP2J2 metabolites (EETs), each of the four regioisomeric forms of EETs (5,6‐EET, 8,9‐EET, 11,12‐EET, and 14,15‐EET) was used to treat cardiomyocytes *in vitro*, and the cells were then stimulated for 24 h with phenylephrine (PE). F‐actin staining (Fig. S3A,B) and RT–PCR analysis (Fig. S3C) revealed that 8,9‐EET, 11,12‐EET, and 14,15‐EET prevented PE‐induced cardiac hypertrophy, but that 11,12‐EET exerted the greatest effect and increased ANP expression more compared with PE stimulation (Fig. S3D,E). Therefore, EETs (especially 11,12‐EET) are responsible for antihypertrophic effect of CYP2J2 overexpression and we used 11,12‐EET to further elucidate the antihypertrophic effects of EETs in the following *in vitro* experiments.

In addition, we found that CYP2J2 overexpression mildly attenuated the hypertensive effect of Ang II in AMPKα2^+/+^ mice (Fig. S4A,B). In order to exclude the effect of blood pressure lowering by CYP2J2 in the development of cardiac hypertrophy, we used hydralazine (100 mg L^−1^) to reduce the blood pressure, the effect of which was consistent with CYP2J2 overexpression (Fig. S5A). We found hydralazine administration exerts weaker antihypertrophic and protective effects than CYP2J2 (Fig. S5B–E and Table S2). Different from CYP2J2, hydralazine did not induce increase in the expression of ANP (Fig. S5F,G). Hydralazine looses smooth muscle cells and therefore lowers blood pressure, without direct roles in cardiomyocytes. However, CYP2J2 overexpression produced both blood pressure lowering and cardiac protection effects. Thus, CYP2J2‐mediated direct cardioprotection was much more than the effect of blood pressure lowering.

### Cardiomyocyte‐specific overexpression of CYP2J2 *in vivo* attenuated myocardial hypertrophy and remodeling via AMPKα2

Previous studies have shown that EETs regulate the phosphorylation, and therefore activation, of 5′‐AMP‐activated protein kinase (AMPK) (Xu *et al*., 2010). We have confirmed CYP2J2 overexpression increased the activity of AMPK in Ang II‐induced cardiac hypertrophy (Fig. S6A–C). To determine whether the antihypertrophic effects of CYP2J2 was through AMPK pathway, rAAV9‐CYP2J2 construct was then injected into the caudal veins of AMPKα2^−/−^ mice, the isoform of which was mainly expressed in heart. Two weeks after injection with rAAV9‐CYP2J2, the mice were exposed to 14 days of continuous infusion of either a saline control or Ang II (1 mg kg^−1^ day^−1^).

**Figure 2:**
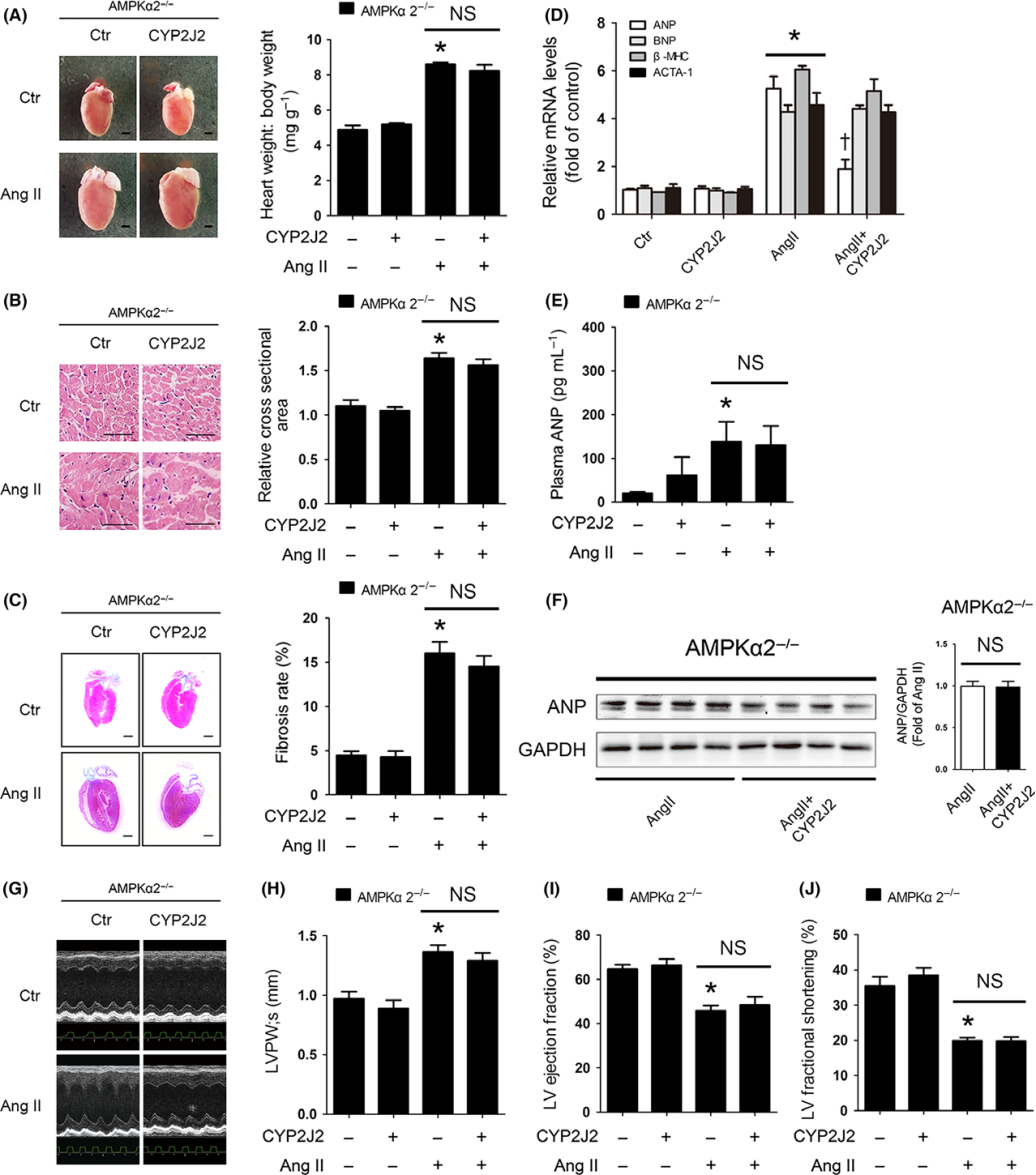
AMPKα2 plays a critical role in CYP2J2‐mediated cardioprotection *in vivo*. AMPKα2^−/−^ mice were injected in the caudal vein with rAAV9‐CYP2J2. After 2 weeks, mice were exposed to continuous infusion of Ang II or a saline control for 14 days (8‐10 mice for each group). (A) Left, the gross morphology of adult hearts from AMPKα2^−/−^ mice 2 weeks after Ang II infusion. Scale bar: 1 mm. Right, heart weight: body weight ratios of adult AMPKα2^−/−^ mice after infusion with Ang II or saline control for 2 weeks. (B) Left, H&E staining of sections of adult hearts from AMPKα2^−/−^ mice after infusion with Ang II or saline control for 2 weeks (Scale bar: 100 μm). Right, quantification of the size of cardiomyocytes by measurement of the cross‐sectional area on H&E‐stained sections. More than 250 cells from three different hearts were analyzed per group. (C) Left, masson trichrome staining of adult hearts from AMPKα2^−/−^ mice after infusion with Ang II or saline control for 2 weeks. The blue area indicates collagen fibers. Scale bar: 1 mm. Right, quantification of the rate of cardiac fibrosis by measurement of the area of collagen deposition. (D) RT–PCR analyses of relative expression of ANP, BNP, β‐MHC, and ACTA1 genes from the hearts of mice exposed to the indicated conditions. (E) ELISA analysis showing the expression of ANP in plasma from AMPKα2^−/−^ mice (*n* = 5–6 for each group). (F) Left, Western blot analyses showing the expression of the ANP protein in Ang II and Ang II+CYP2J2 groups. GAPDH was used as a loading control. Right, the intensity of the Western blot signal was quantified and is shown as relative protein expression after normalization to GAPDH. (G) Representative images of echocardiograms. (H) LVPW;s. (I) LV ejection fraction. (J) LV fractional shortening. The data represent the mean ± SEM from at least four independent experiments (*P < 0.05 vs. control group; ^†^ *P* < 0.05 vs. Ang II group).

Interestingly, the cardioprotection effect of CYP2J2 overexpression in AMPKα2^+/+^ mice was not observed in AMPKα2^−/−^ mice. Ang II‐induced increased heart size (Fig. 2A), area of the cardiomyocytes (Fig. 2B), fibrosis of cardiomyocytes (Fig. 2C), and biomarkers of cardiac hypertrophy (Fig. 2D) were not attenuated after CYP2J2 overexpression in AMPKα2^−/−^ mice. Additionally, increased ANP expression in CYP2J2‐mediated cardioprotection was also not seen in AMPKα2^−/−^ mice (Fig. 2E,F). Echocardiography results showed CYP2J2 overexpression plays little role against Ang II‐induced cardiac hypertrophy in AMPKα2^−/−^ mice (Fig. 2G–J and Table S3).

Thus, these data together demonstrated that cardiomyocyte‐specific overexpression of CYP2J2 *in vivo* attenuated myocardial hypertrophy and remodeling partially via AMPKα2.

### 11,12‐EET inhibited the hypertrophic response of cardiomyocytes by increasing ANP expression in an AMPKα2‐dependent manner

**Figure 3:**
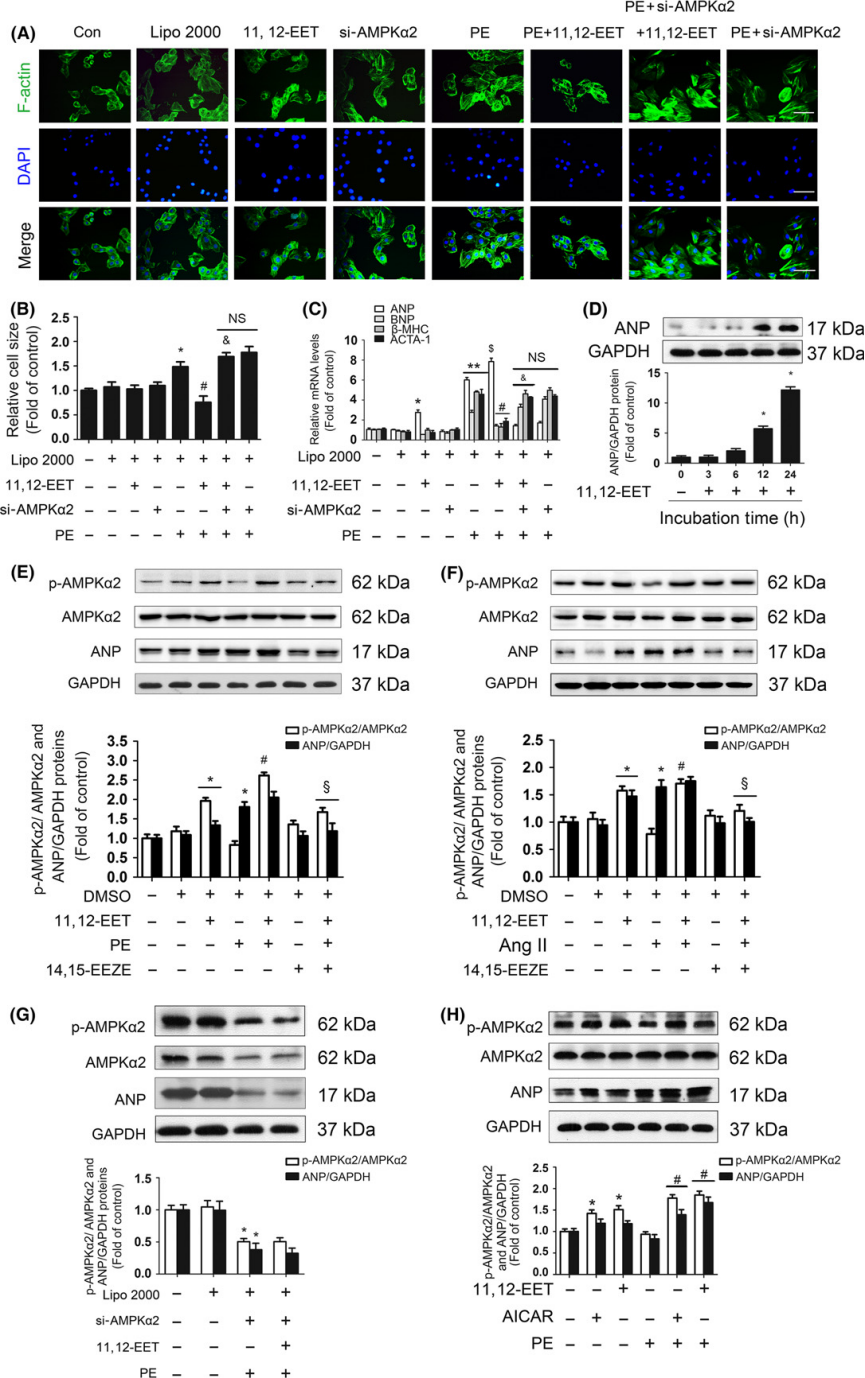
11,12‐EET inhibited the hypertrophic response of cardiomyocytes by increasing ANP expression in a manner dependent on AMPKα2 phosphorylation. (A) Adult mouse primary cardiomyocytes were transfected with AMPKα2 siRNA (100 nmol L^−1^), treated with 11,12‐EET (1 μmol L^−1^), and then stimulated with PE (50 μmol L^−1^) for 24 h. Representative images of cells from different groups treated as described above and immunostained for f‐actin (green) and for the nuclear marker DAPI (blue) (Scale bar: 100 μm). (B) Quantification of the size of mouse cardiomyocytes for each group (25 cells/condition in each preparation; four independent preparations). (C) RT–PCR analyses of the relative expression of ANP, BNP, β‐MHC, and ACTA1 in mouse primary cardiomyocytes subjected to the indicated treatments. (D) Analyses of ANP protein expression in a time‐dependent manner by Western blotting. (E) Phosphorylation of AMPKα2 and expression of ANP in response to PE (50 μmol L^−1^) stimulation in the presence of 14,15‐EEZE (1 μmol L^−1^), shown by Western blotting. (F) Phosphorylation of AMPKα2 and expression of ANP in response to Ang II stimulation (1 μmol L^−1^) in the presence of 14,15‐EEZE (1 μmol L^−1^). (G) Analyses of p‐AMPKα2 and ANP protein expression by Western blotting. (H) Analyses of p‐AMPKα2 and ANP protein expression by Western blotting after PE (50 μmol L^−1^) stimulation in the presence of 11,12‐EET (1 μmol L^−1^) or AICAR (1 μmol L^−1^). The data represent the mean ± SEM from at least four independent experiments. (*P < 0.05 vs. control; **P < 0.01 vs. control; ^$^ *P* < 0.05 vs. control; ^#^ *P* < 0.05 vs. PE group; ^&^ *P* < 0.05 vs. PE+11,12‐EET group; ^§^ *P* < 0.05 vs. PE+11,12‐EET or Ang II+11,12‐EET group).

To determine whether the protective effect of 11,12‐EET against the development of hypertrophy was also mediated by the activation of AMPKα2, cardiomyocytes were transfected with AMPKα2 siRNA, treated with 11,12‐EET, and treated with PE. As expected, PE stimulation significantly produced cardiac hypertrophy, which was associated with an increased size of cardiomyocytes (Fig. 3A,B) and increased mRNA levels of BNP, β‐MHC, and ACTA1 (Fig. 3C). Pretreatment with 11,12‐EET markedly attenuated these PE‐induced changes. However, this effect was abrogated in the presence of AMPKα2 siRNA (Fig. 3A–C). In accordance with the *in vivo* results, 11,12‐EET treatment increased levels of ANP mRNA (Fig. 3C) and protein (Fig. 3D–F) expression in a time‐dependent manner (Fig. 3D). 11,12‐EET treatment increased phosphorylation of AMPKα2 under either baseline or PE‐ (Fig. 3E) or Ang II stimulation (Fig. 3F)‐induced cardiac hypertrophy. And these effects of 11,12‐EET on phosphorylation of AMPKα2 were associated with expression of ANP (Fig. 3E,F). However, 11,12‐EET‐induced phosphorylation of AMPKα2 and expression of ANP were not observed after adding EET antagonist 14,15‐EEZE. Additionally, 11,12‐EET did not induce overexpression of ANP in the presence of AMPKα2 siRNA (Fig. 3G). Given PE or Ang II stimulations produced similar effects on phosphorylation of AMPKα2 and the PE stimulations were more stable than Ang II, we choose PE to take the *in vitro* experiments. In addition, the AMPK agonist 5‐aminoimidazole‐4‐carboxamide ribonucleotide (AICAR) also upregulated ANP expression after PE challenges (Fig. 3H). These data suggest that 11,12‐EET protects against PE‐ or Ang II‐induced cardiac hypertrophy by activating AMPKα2 and consequently increasing levels of ANP.

### CYP2J2 overexpression or 11,12‐EET administration triggered AMPKα2‐dependent nuclear translocation of p‐Akt1

**Figure 4:**
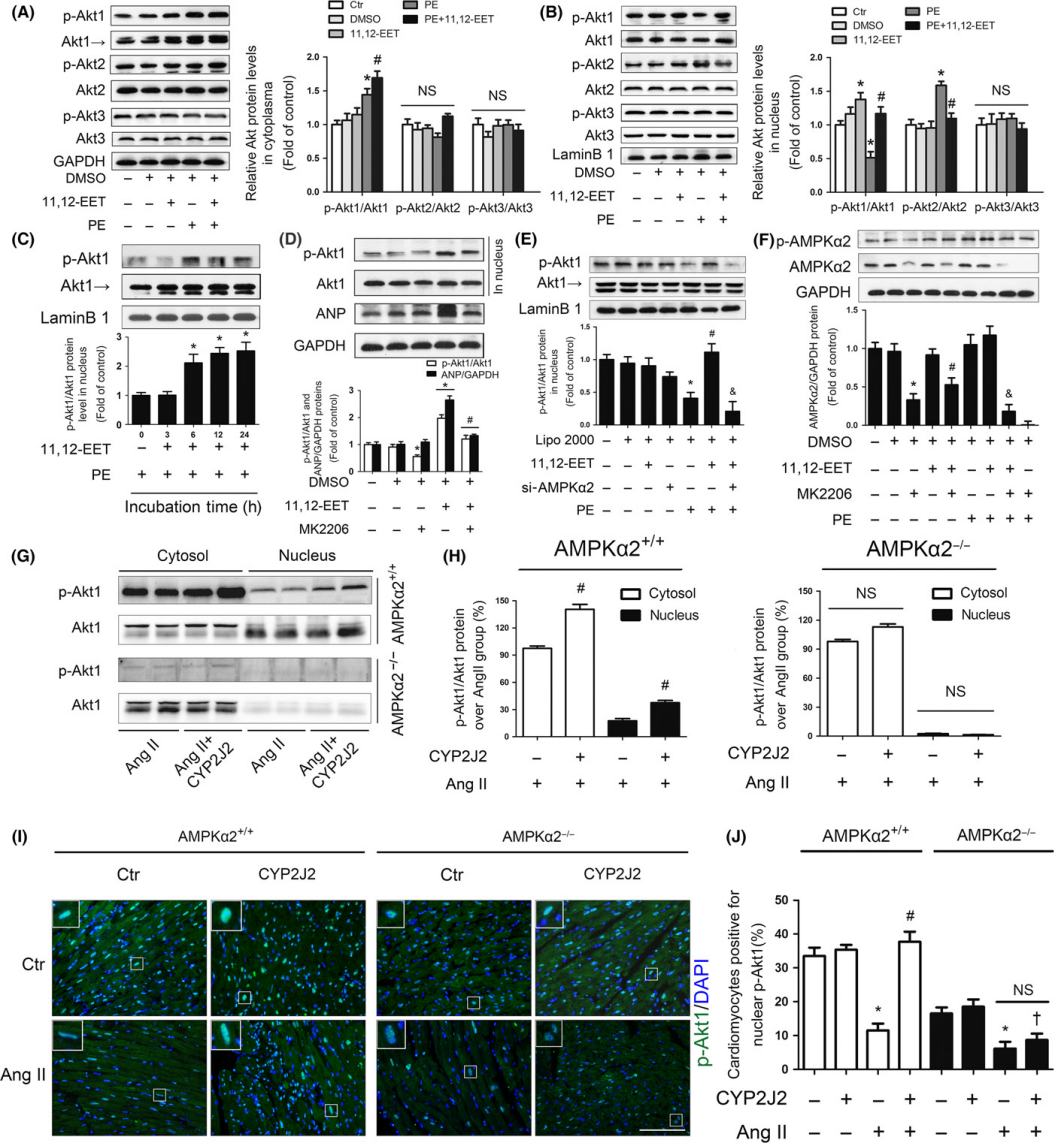
CYP2J2 overexpression or 11,12‐EET administration triggered p‐Akt1 nuclear translocation through AMPKα2 effects. (A) Left, Western blot analysis showing the phosphorylation of Akt1, Akt2, and Akt3 in the cytosol of mouse cardiomyocytes exposed to PE with or without 11,12‐EET pretreatment. GAPDH expression was determined to validate equal sample loading. The blots shown are representative of four independent experiments. Right, quantitative analysis of gray density of Western blotting bands from four independent experiments. (B) Left, Western blot analysis showing the phosphorylation of Akt1, Akt2, and Akt3 in the nucleus of mouse cardiomyocytes exposed to PE with or without 11,12‐EET pretreatment. Lamin B1 expression was determined to validate equal sample loading. The blots shown are representative of four independent experiments. Right, quantitative analysis of the gray density of Western blotting bands from four independent experiments. (C) Analyses of expression of the nuclear p‐Akt1 protein in a time‐dependent manner by Western blotting. (D) Western blotting analyses showing the expression of ANP and p‐Akt1 in the nucleus. (E) Western blotting analyses of nuclear p‐Akt1 in the presence of AMPKα2 siRNA. (F) Western blotting analyses of p‐AMPKα2 and AMPKα2 in the presence of the Akt1‐selective inhibitor MK2206 (8 nmol L^−1^). (G) Immunoblots of heart tissue homogenates from the indicated strains of mice in which antibodies specific for p‐Akt1 (Thr473) and total Akt1 were used. Mice hearts were collected after infusion with Ang II or a saline control for 2 weeks. The results are representative of four independent experiments with four mice hearts per group. (H) Left, quantitative analysis of the gray density of Western blotting bands from AMPKα2^+/+^ mice; Right, quantitative analysis of the gray density of Western blotting bands from AMPKα2^−/−^ mice. CYP2J2 overexpression increased phosphorylation of Akt1 (Ser473) compared with Ang II infusion in AMPKα2^+/+^ mice heart tissues, the effect of which was not observed in AMPKα2^−/−^ mice and accumulation of p‐Akt1 (Ser473) in nucleus was AMPKα2 dependent. (I) Immunofluorescence staining with the p‐Akt1 antibody (green) and DAPI (blue) of transverse sections of adult hearts from AMPKα2^+/+^ and AMPKα2^−/−^ mice (Scale bar: 100 μm). (J) The number of cardiomyocytes positive for nuclear p‐Akt1 was counted in four different mice, with a total of 800 cardiomyocytes counted in each group. All data represent the mean ± SEM from at least four independent experiments (*P < 0.05 vs. corresponding control; ^#^ *P* < 0.05 vs. PE group *in vitro* and vs. Ang II *in vivo*; ^&^ *P* < 0.05 vs. PE+11,12‐EET group; ^†^ *P* < 0.05 vs. Ang II+CYP2J2 group of AMPKα2^+/+^ mice).

Previous studies demonstrated that the phosphatidyl inositol 3‐kinase–Akt signaling pathway protects the heart against injuries and is therefore called a survival factor for cardiomyocytes; however, this pathway also induces cardiac hypertrophy (Fujio *et al*., 2000; Condorelli *et al*., 2002; Zhao *et al*., 2015). Our previous study demonstrated that EETs and CYP epoxygenases markedly activate Akt and inhibit cardiac hypertrophy (Xiao *et al*., 2010). To further understand this controversial phenomenon, we investigated whether 11,12‐EET‐induced ANP expression was mediated by the activation of Akt in cardiac hypertrophy and whether EETs enhanced the nuclear translocation of Akt. First, we incubated cardiomyocytes with 11,12‐EET and then subjected to PE treatment for 24 h. Pretreatment with 11,12‐EET resulted in increased phosphorylation of Akt1 in cytosol after PE stimulation, but there were no significant effects on the phosphorylation of Akt2 or Akt3 (Fig. 4A). PE stimulation resulted in reduced p‐Akt1 levels in nucleus, but pretreatment with 11,12‐EET prevented this phenomenon. Pretreatment with 11,12‐EET did not result in increased p‐Akt2 and p‐Akt3 levels in the nucleus in cardiomyocytes that had undergone PE stimulation. On the contrary, 11,12‐EET reduced p‐Akt2 level in the nucleus in cardiomyocytes after PE stimulation (Fig. 4B). These findings indicate that 11,12‐EET induced an accumulation of p‐Akt1 in nucleus, which prevented the reduction in p‐Akt1 levels observed during PE‐induced hypertrophy. To confirm this effect of 11,12‐EET on p‐Akt1, time‐dependent experiments were performed and 11,12‐EET pretreatment was shown to result in an accumulation of p‐Akt1 in the nucleus 6 h after PE stimulation (Fig. 4C).

Notably, 11,12‐EET‐induced expression of ANP was not observed in the presence of the Akt1‐selective inhibitor MK2206 (Fig. 4D), which suggests that the accumulation of activated Akt1 in the nucleus was required for 11,12‐EET‐induced expression of ANP. Given that the increased expression of ANP induced by 11,12‐EET required both the activation of AMPKα2 and the nuclear accumulation of active Akt1, we speculated that these two molecules interacted. In the presence of AMPKα2 siRNA, 11,12‐EET‐induced accumulation of activated Akt1 in nucleus was prevented (Fig. 4E). Interestingly, in the presence of MK2206, the phosphorylation of AMPKα2 was not significantly affected, but the expression of AMPKα2 was significantly decreased (Fig. 4F). These data show the existence of cross talk between AMPKα2 and nuclear p‐Akt1.

In accordance with the results *in vitro*, Western blots of cardiac tissue from AMPKα2^+/+^ mice showed that p‐Akt1 accumulation in the nucleus was reduced after Ang II infusion *in vivo*, but accumulation of p‐Akt1 was increased in the cytosol (Fig. S7A–C), suggesting that Ang II leads to the export of p‐Akt1 from the nucleus to the cytosol. These findings were not observed in AMPKα2^−/−^ mice. CYP2J2 overexpression attenuated Ang II‐induced reductions in nuclear p‐Akt1 levels in AMPKα2^+/+^ mice, but not in AMPKα2^−/−^ mice (Fig. 4G,H). Staining of cardiomyocytes for p‐Akt1 also depicted that forced expression of CYP2J2 prevented the reduction in nuclear p‐Akt1 levels that was observed after Ang II stimulation in AMPKα2^+/+^ mice; forced CYP2J2 expression did not have this effect in AMPKα2^−/−^ mice (Fig. 4I,J).

Taken together, these results indicate that CYP2J2 or its metabolite 11,12‐EET induced the nuclear accumulation of p‐Akt1 via AMPKα2 to prevent development of cardiac hypertrophy.

### 11,12‐EET increased the nuclear translocation of p‐Akt1 by affecting the binding of AMPKα2β2γ1 to p‐Akt1

**Figure 5:**
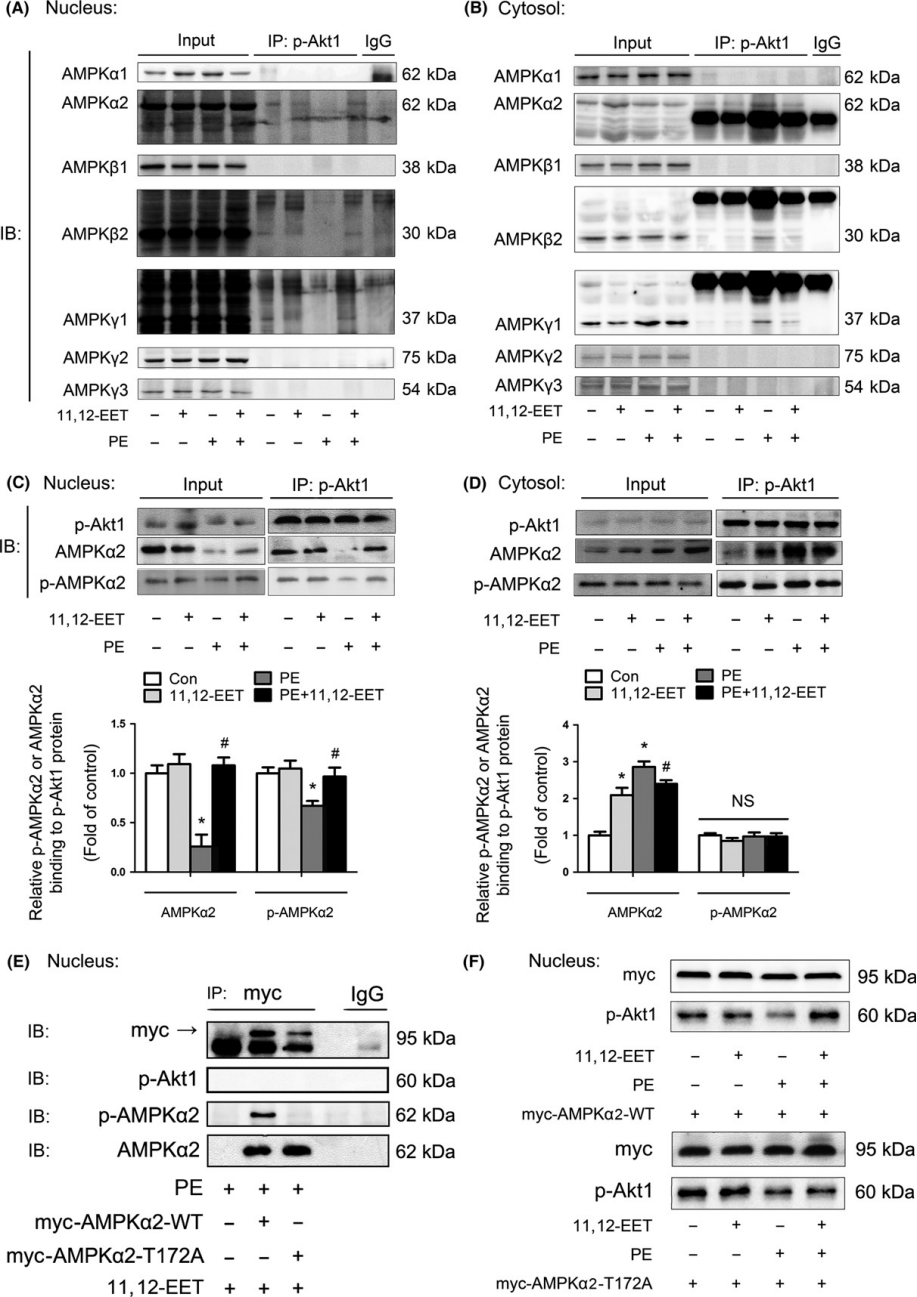
11,12‐EET increased the nuclear translocation of p‐Akt1 by affecting the binding of AMPKα2β2γ1 to p‐Akt1. (A, B) Immunoprecipitation assays testing the interactions between p‐Akt1 and proteins encoded by seven AMPK subunit‐related genes in cultured mouse cardiomyocytes. Nuclear extracts (A) and cytosol preparations (B) of mouse cardiomyocytes were obtained from different groups. The lysates were extracted for immunoprecipitation with a p‐Akt1‐specific antibody or a control IgG, which was followed by probing with antibodies specific for AMPKα1, AMPKα2, AMPKβ1, AMPKβ2, AMPKγ1, AMPKγ2, and AMPKγ3. The blots shown are representative of four independent experiments. (C, D) Immunoprecipitation assays testing the interactions between p‐Akt1 and AMPKα2. Nuclear extracts (C) and cytosol preparations (D) of mouse cardiomyocytes were obtained after PE stimulation from different groups. The nuclear lysates were extracted for immunoprecipitation with a p‐Akt1‐specific antibody, which was followed by blotting with antibodies specific for p‐AMPKα2 and AMPKα2. (E) The effect of the AMPKα2 T172A mutation on the interaction between p‐Akt1 and p‐AMPKα2, and between p‐Akt1 and AMPKα2, with the presence of 11,12‐EET and PE. HEK293T cells were transfected with myc‐AMPKα2‐WT and myc‐AMPKα2‐T172A. The nuclear lysates were extracted for immunoprecipitation with a myc‐specific antibody or a control IgG, which was followed by blotting with antibodies for p‐Akt1, p‐AMPKα2, and AMPKα2. (F) Top, HEK293T cells were transfected with myc‐AMPKα2‐WT plasmid with or without the presence of 11,12‐EET and PE. Western blotting analyses show the expression of nuclear p‐Akt1. Bottom, HEK293T cells were transfected with the myc‐AMPKα2‐T172A plasmid with or without the presence of 11,12‐EET and PE. Western blotting analyses show the expression of nuclear p‐Akt1. The data represent the mean ± SEM from at least four independent experiments (*P < 0.05 vs. corresponding control; ^#^ *P* < 0.05 vs. PE group).

To further investigate the interaction between AMPKα2 and p‐Akt1, immunoprecipitation experiments were conducted. p‐Akt1 directly interacted with the AMPKα2β2γ1 isoform both in nucleus (Fig. 5A) and in cytosol (Fig. 5B). Moreover, in cardiomyocytes stimulated with PE, the amount of AMPKα2β2γ1 bound to p‐Akt1 was reduced in nucleus, but increased in cytosol. Pretreatment of PE‐stimulated cardiomyocytes with 11,12‐EET increased the amount of AMPKα2β2γ1 bound to p‐Akt1 in nucleus (Fig. 5A) but reduced the amount of AMPKα2β2γ1 bound to p‐Akt1 in cytosol (Fig. 5B). These results suggest that 11,12‐EET protected against PE‐induced cardiac hypertrophic response by inducing an influx of AMPKα2β2γ1‐bound p‐Akt1 from cytosol to nucleus.

Both p‐AMPKα2 (Thr172), in which the Thr172 amino acid residue is phosphorylated, and AMPKα2 interacted with p‐Akt1 (Fig. 5C,D). To determine whether the effect of 11,12‐EET was dependent on the phosphorylation of AMPKα2 (Thr172), a myc‐conjugated AMPKα2‐T172A mutated form was constructed to remove the phosphorylation site, which was then used for transfection of HEK293T cells. In cells transfected with myc‐conjugated AMPKα2‐WT, but not in cells transfected with myc‐conjugated AMPKα2‐T172A, 11,12‐EET prevented the reduction in nuclear accumulation of p‐Akt1 that was observed with PE stimulation (Fig. 5F). These findings indicate that the nuclear accumulation of p‐Akt1 required the phosphorylation of AMPKα2 at Thr172. However, immunoprecipitation experiments of nuclear extracts indicated that the 11,12‐EET‐induced association between p‐Akt1 and AMPK did not occur with either myc‐conjugated AMPKα2‐WT or myc‐conjugated AMPKα2‐T172A (Fig. 5E), which suggests that the p‐Akt1 binding site was not in the α2 subunit of AMPKα2β2γ1. Therefore, we speculated that this binding process was separate from nuclear translocation of p‐Akt1.

### 11,12‐EET induced the γ1 subunit of AMPKα2β2γ1 to bind with p‐Akt1 protein kinase domain, which subsequently led to nuclear translocation of p‐Akt1

**Figure 6:**
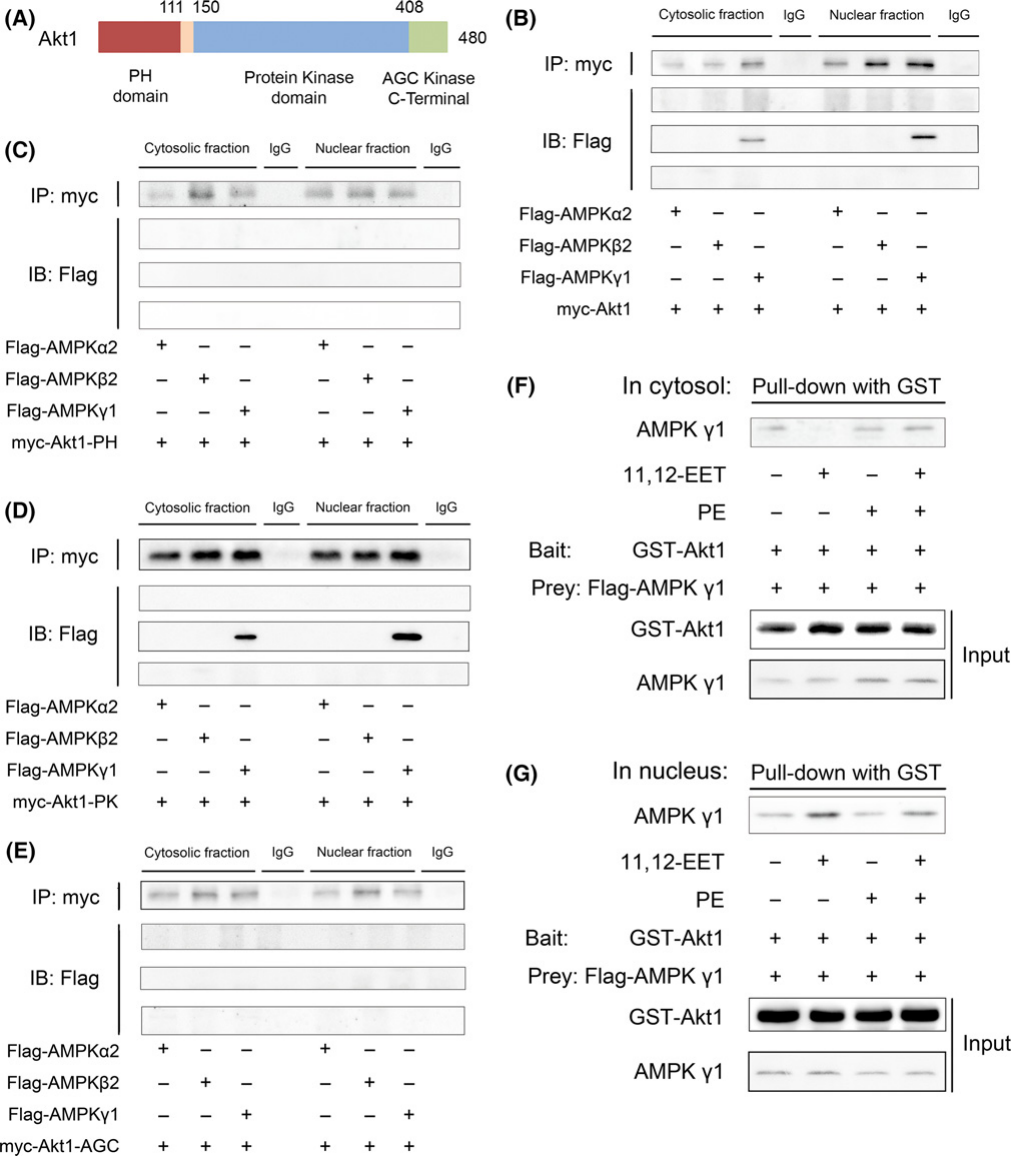
11,12‐EET induced the γ1 subunit of AMPKα2β2γ1 to bind with the p‐Akt1 protein kinase domain, which subsequently led to nuclear translocation of p‐Akt1. (A) Akt1 domains. (B) Full‐length Akt1 tagged with myc (myc‐Akt1), (C) an Akt1 fragment containing amino acids 4–111, (D) an Akt1 fragment containing amino acids 150–408 (myc‐Akt1‐PK), and (E) an Akt1 fragment containing amino acids 409–479 (myc‐Akt1‐AGC) were incubated with Flag‐tagged AMPKα2, AMPKβ2, or AMPKγ1 in HEK293T cells. (F, G) HEK293T cells were transiently transfected with GST‐Akt1 for 24 h using Lipo2000, pretreated with 11,12‐EET, and then subjected to PE stimulation for 24 h. After incubation, extracted (F) cytosolic and (G) nuclear proteins were purified with glutathione Sepharose beads. The precipitates and lysates were individually immunoblotted with antibodies against Akt1 or AMPKγ1. All data represent at least four independent experiments.

To determine which part of AMPKα2β2γ1 bound with which part of p‐Akt1, three Akt1 fragments of the protein containing distinct domains were constructed according to the molecular structure of Akt1 (Fig. 6A). An *in vitro* binding assay showed that full‐length Akt1 (Fig. 6B) and an Akt1 fragment containing amino acids 150–408, which constitute the protein kinase domain (Fig. 6D), but not other fragments of Akt1 (Fig. 6C,E), bind to the AMPKγ1 subunit. None of the Akt fragments were found to bind to AMPKα2 or AMPKβ2. A glutathione *S*‐transferase (GST) pull‐down assay revealed that 11,12‐EET increased the binding of AMPKγ1 to Akt1 in the nucleus but not in the cytosol (Fig. 6F,G). Together, these results suggest that 11,12‐EET promoted the nuclear translocation of p‐Akt1 by enhancing the interaction between the AMPKγ1 subunit directly with Akt1 protein kinase domain.

## Discussion

In this study, we provide the evidences that cardiomyocyte‐specific forced expression of CYP2J2 attenuated cardiomyocyte hypertrophy *in vivo* through the activation of AMPKα2. Mechanistically, we found that CYP2J2‐derived EETs triggered AMPKα2β2γ1 binding to the p‐Akt1 protein kinase domain, which was accompanied by nuclear translocation of p‐Akt1. This process, in turn, increased the expression of ANP. Thus, CYP2J2, EETs, and downstream signaling molecules may be novel therapeutic targets for protection against the development of cardiac hypertrophy and consequential heart failure. Our proposed model for the mechanism by which AMPKα2 mediates the protective effect of CYP2J2/11,12‐EET on cardiac hypertrophy is summarized in Fig. S8.

Cardiac hypertrophy and consequential heart failure occur more frequently in older population. In a recent population‐based study in US, the prevalence of heart failure was 2.2% (95 CI 1.6–2.8%), increasing from 0.7% in persons aged 45 through 54 years to 8.4% for those aged 75 years or older (Redfield *et al*., 2003). Neurohormonal dysregulation (such as increased Ang II levels) in aged heart may somehow explain why the 5‐year risk rate of cardiac hypertrophy and heart failure was higher in older persons. In our current study, CYP2J2 overexpression significantly prevented Ang II‐induced cardiac hypertrophy, and therefore, we speculated CYP2J2 may have important protective effects against aging‐induced cardiac hypertrophy and consequential heart failure.

Previous studies have demonstrated that increasing the levels of EETs by inhibiting soluble epoxide hydrolase confers cardioprotection against Ang II‐induced cardiac hypertrophy (Ai *et al*., 2009), but the mechanisms of this action were unclear. In this study, we investigated the protective effect of CYP2J2 in Ang II‐infused mice and the effect of exogenous EETs on PE‐induced model of cellular hypertrophy. AMPKα2 deficiency exacerbates pressure overload‐induced left ventricular hypertrophy and cardiac dysfunction in mice (Zhang *et al*., 2008) and we demonstrated that exogenous EET treatment activates AMPK in mouse heart tissue (Ma *et al*., 2013). Thus, it was reasonable to postulate that AMPKα2 activation might mediate the protective effects of CYP2J2 overexpression or EETs against the development of cardiac hypertrophy. In our current study, overexpression of CYP2J2 attenuated cardiac hypertrophy in AMPKα2^+/+^ mice that underwent Ang II infusion, but not in AMPKα2^−/−^ mice similarly treated. Furthermore, the antihypertrophic effect of 11,12‐EET was abolished after transfection with AMPKα2 siRNA. These findings indicate that the protective effect of CYP2J2 or EETs against the development of cardiac hypertrophy was dependent on AMPKα2.

Hallmarks of cardiac hypertrophy include increased myocardial cell size, increased sarcomeric organization, re‐activation of genes with a typical fetal expression (i.e. ANP, BNP, β‐MHC), and enhanced protein synthesis (Chien *et al*., 1993; Wang *et al*., 2013). In our mouse and cell models of cardiac hypertrophy, myocardial cell size and the levels of BNP, β‐MHC, and ACTA1 were all increased after exposure to Ang II or PE. However, the transcriptional and translational levels of ANP were regulated by the activation of AMPKα2. We, therefore, focused our attention on the relationship between AMPKα2 and ANP in cardiac hypertrophy. ANP has numerous biological actions that inhibit hypertrophy, including diuretic, natriuretic, and vasodilatory effects, as well as autocrine and paracrine actions on cardiomyocytes (Nishikimi *et al*., 2006; Rubattu *et al*., 2008; Horikawa *et al*., 2011). The increase in ANP that occurs with cardiac hypertrophy might help compensate for the increase in afterload. In the past few years, studies have revealed that the antihypertrophic effect of ANP is mediated by the guanylylcyclase‐A receptor and cGMP production (Klaiber *et al*., 2011; Lee *et al*., 2015). Furthermore, ANP has been used to reduce cardiac remodeling after myocardial infarction (Kuga *et al*., 2003). Thus, we speculate that CYP2J2 and EETs might regulate the expression of ANP through the activation of AMPKα2 to attenuate cardiac hypertrophy.

Several studies demonstrated that Akt localization in the nucleus prevented cardiac hypertrophy and maintained cardiac function following thoracic aorta constriction (TAC) by increasing ANP expression (Tsujita *et al*., 2006; Horikawa *et al*., 2011). Nuclear targeting of Akt also enhances kinase activity and the survival of cardiomyocytes (Shiraishi *et al*., 2004), and 17‐estradiol can attenuate cardiac hypertrophy by activating Akt1 (through phosphorylation at Ser473), which increases ANP production (Camper‐Kirby *et al*., 2001). Besides enhancing nuclear translocation of Akt1, CYP2J2 or EETs may also protect against lipopolysaccharide‐induced cardiac dysfunction via ablation of Akt2 (Zhang *et al*., 2014). Our previous study suggested that either overexpression of CYP2J2 or exogenous EET increased the expression of Akt (Wang *et al*., 2005). Therefore, we speculated that EET increased nuclear Akt translocation, which triggered the expression of ANP, thereby inhibiting cardiac hypertrophy. As ANP was regulated both by the activation of AMPKα2 and by nuclear p‐Akt1 in our current study, we sought to determine whether a physical interaction exists between AMPKα2 and p‐Akt1 and whether this interaction leads to translocation of p‐Akt1 to the nucleus. We found that AMPKα2β2γ1 binds to p‐Akt1 (Ser473) and that this binding was dependent on the phosphorylation of AMPKα2 (at Thr172). However, the ability of AMPKα2 to bind to p‐Akt1 was abolished after transfection with AMPKα2‐WT or AMPKα2‐T172A in a HEK293T cell line, indicating that physical interaction between p‐Akt1 and AMPK did not involve the AMPKα2 subunit. As AMPK possesses one catalytic (α) and two regulatory (β and γ) subunits, we then assessed whether the β or γ subunit contributed to this process. Our results indicated that the protein kinase domain of Akt1, but not other fragments of this protein, could bind to the AMPKγ1 subunit. In addition, 11,12‐EET promoted the nuclear translocation of Akt1 by enhancing the interaction between AMPKγ1 with the Akt1 protein kinase domain.

Overall, our data indicate that CYP2J2 and its metabolites EETs inhibited cardiac hypertrophy through the activation of AMPKα2. Importantly, CYP2J2 and EET induced the formation of the AMPKα2β2γ1–pAkt1 complex, leading to the nuclear translocation of p‐Akt1, which, in turn, upregulated the expression of ANP, attenuating cardiac hypertrophy. Our data define the important relationship between CYP2J2 and AMPKα2 and identify a novel mechanism for the antihypertrophic effects of these proteins. The study provides a therapeutic rationale for the potential of increasing CYP2J2 expression or EET levels to increase the levels of AMPKα2 and its beneficial effects against heart failure.

## Experimental procedures

### Genetically modified mice

AMPKα2 cardiac myocyte‐specific knockout (AMPKα2^−/−^) mice were obtained as a gift from Dr Ming‐Hui Zou (University of Oklahoma Health Science Center, Oklahoma City, Oklahoma). AMPKα2^−/−^ mice and their genetic controls (AMPKα2^+/+^ mice) were bred at the animal care facility of Tongji Medical College under specific pathogen‐free conditions. Mice were housed in temperature‐controlled cages under a 12‐h light–dark cycle and given free access to water and normal chow. Age‐matched 8‐ to 10‐week‐old male AMPKα2^−/−^ mice and littermates were used, and all experiments were conducted using procedures approved by Institutional Animal Care in accordance with the NIH Guide for the Care and Use of Laboratory Animals.

### Recombinant adeno‐associated virus Serotype 9 (rAAV9)‐mediated gene transfer

The rAAV vectors (Serotype 9) containing CYP2J2 was produced by triple plasmid cotransfection in HEK293 cells as previously described (Jiang *et al*., 2007). Purified rAAV9‐CYP2J2 (1 × 10^11^ pfu) was injected into the caudal vein of AMPKα2^−/−^ and AMPKα2^+/+^ mice 14 days prior to Ang II infusion. Mice were first anesthetized using an intraperitoneal injection of 1% sodium pentobarbital. A 29‐gauge sterile needle and syringe were then used to deliver virus in a volume of 150 μL.

### Ang II infusion

Ang II (1 mg kg^−1^ day^−1^) (Sigma‐Aldrich, St. Louis, MO, USA) or saline vehicle was infused via osmotic minipumps (Model 1002; Alzet, Cupertino, CA, USA) that were implanted subcutaneously under 1% sodium pentobarbital anesthesia in AMPKα2^−/−^ and AMPKα2^+/+^ mice. Osmotic pumps containing saline were used as controls. After 14 days, mice were subjected to transthoracic echocardiography and cardiac catheterization to determine cardiac function. Blood pressure was measured by tail‐cuff as described previously (Chamorro‐Jorganes *et al*., 2010). Animals were habituated to the blood pressure apparatus for several days before the data were collected. Ten to fifteen repeated values of systolic blood pressure were averaged at each determination.

### Hydralazine administration

After rAAV9‐mediated gene transfer and Ang II infusion, a part of wild‐type and AMPKα2^−/−^ mice with Ang II infusion were treated with/without various concentrations of hydralazine (Sigma‐Aldrich) in drinking water (200, 150, 100, and 50 mg L^−1^). After 14 days, mice were subjected to cardiac catheterization to determine cardiac function. Blood pressure was measured by tail‐cuff as described previously (Chamorro‐Jorganes *et al*., 2010).

### Cell culture and treatment

Primary cultures of adult mouse cardiomyocytes were prepared as described previously (Zhou *et al*., 2000). Collected cells were cultured in Dulbecco's modified Eagle's medium (Life technologies, Cergy‐Pontoise, France) containing 4.5 g L^−1^ glucose, 1.5 g L^−1^ sodium bicarbonate, and 110 mg L^−1^ sodium pyruvate, supplemented with 10% fetal bovine serum (Gibco, New Zealand) and penicillin (100 units mL^−1^) and streptomycin (100 mg mL^−1^) in a humidified incubator with 95% air and 5% CO2 at 37°C. The culture medium was changed every day. Then, the cells were passaged and seeded at the density of 0.4 × 10^6^ cells per 34.8 mm well of 6 well plates or 3 × 10^4^ cells per 15.6 mm well of 24 well plates. These cells were cultured for 2–3 days and then underwent treatments. Cardiomyocytes were transfected with AMPKα2 siRNA (100 nmol L^−1^) or Akt1‐selective inhibitor MK2206 (8 nmol L^−1^) for 1 h and then with 11, 12‐EET (1 μmol L^−1^) (Cayman Chemical, Ann Arbor, MI, USA) for another 1 h prior to phenylephedrine (PE) treatment. PE (50 μmol L^−1^) (Sigma‐Aldrich) was prepared in double‐distilled water and diluted with culture media to induce hypertrophy and cultured for an additional 24 h (Xia *et al*., 2004). The experimental group consists of (i) control cells, (ii) Lipo 2000 alone treated cells, (iii) 11, 12‐EET alone treated cells, (iv) si‐AMPKα2 alone treated cells (or MK2206 alone treated cells), (v) PE alone treated cells, (vi) PE+11, 12‐EET treated cells, (vii) PE+11, 12‐EET+ si‐AMPKα2 (or MK2206) treated cells, (viii) PE+si‐AMPKα2 (or MK2206) treated cells. By adding EET antagonist 14,15‐EEZE (1 μmol L^−1^), experimental groups were (i) control, (ii) DMSO, (iii) 11, 12‐EET alone, (iv) PE (Ang II, 1 μmol L^−1^) alone, (v) PE (Ang II) + 11,12‐EET, (vi) 14,15‐EEZE, (vii) PE (Ang II) + 11,12‐EET + 14,15‐EEZE.

In some experiments, HEK 293T cells (American Type Culture Collection, ATCC, P.O. Box 1549, Manassas, Va. 20108, USA) were used. Cultured HEK 293T cells were used for plasmids transfection. All Western blotting, immunoprecipitations, and phosphorylation experiments were performed as described previously (Westphal *et al*., 1999).

### Statistical analysis

All data are reported as the mean ± SEM. Statistical analyses between groups were performed with unpaired Student's *t*‐test or one‐way analysis of variance followed by a post hoc Fisher's comparison test. A *P* value of < 0.05 was considered significant.

Note: Experimental details for antibodies and reagents, echocardiographic analysis, hemodynamic measurements of left ventricular function, cell area measurement, plasmid constructs and transfection, nuclear extract and cytosol preparation, immunohistochemical analysis, co‐immunoprecipitation and GST pull‐down, apoptosis assay, and RT–PCR are included in the Appendix S1.

## Funding

This work was supported by National Natural Science Foundation Committee key project (No. 31130031) and NSFC Vessel Research Plan project (No. 91439203).

## Author contributions

Bei Wang designed and performed all the animal and *in vitro* experiments and wrote the manuscript draft; Hesong Zeng and Zheng Wen helped in part of the experiments; and Chen Chen was involved in the designment and discussed the results. Dao Wen Wang, corresponding author, provided financial supports, designed the study, and finished final writing of the manuscript.

## Conflict of interest

The authors declare that they have no competing interests.

## Supporting information


**Fig. S1** P450 epoxygenase overexpression mediated by rAAV9 and quantitative analysis of 11,12‐EET and 11,12‐DHET in cardiac tissues.

**Fig. S2** Cardiomyocyte‐specific overexpression of CYP2J2 maintained cardiac function.

**Fig. S3** Different effects of the four regioisomeric forms of EET on PE‐induced cardiac hypertrophy *in vitro*.

**Fig. S4** Overexpression of CYP2J2 mildly attenuated systolic blood pressure and increased ANP expression after Ang II infusion in AMPKα2^+/+^ mice.

**Fig. S5** Overexpression of CYP2J2 in cardiomyocytes exerts stronger antihypertrophic effects than administration of hydralazine in AMPKα2^+/+^ mice.

**Fig. S6** Overexpression of CYP2J2 in cardiomyocytes increased the activity of AMPK compared with AngII in AMPKα2^+/+^ mice.

**Fig. S7** Cytoplasmic and nuclear Akt1 expression in AMPKα2^+/+^ and AMPKα2^−/−^ heart extracts in basal state and after Ang II treatment.

**Fig. S8** Proposed model for the signaling pathway by which CYP2J2 or 11,12‐EET attenuates cardiac hypertrophic response.

**Table S1** Primers for quantitative real‐time PCR.

**Table S2** Hemodynamic characteristics of animal groups.

**Table S3** Echocardiographic characteristics of animal groups.

**Appendix S1** Extended Experimental Procedures.
