# Supplementary material for: CYP2J2 and its metabolites (epoxyeicosatrienoic acids) attenuate cardiac hypertrophy by activating AMPKα2 and enhancing nuclear translocation of Akt1
Source: Aging Cell. 2016 Jul 14;15(5):940–52. doi: 10.1111/acel.12507 (PMC5013012; doi:10.1111/acel.12507)
Supplement: Supplementary file 1 — Fig. S1 P450 epoxygenase overexpression mediated by rAAV9 and quantitative analysis of 11,12‐EET and 11,12‐DHET in cardiac tissues. [file ACEL-15-940-s001.pdf]

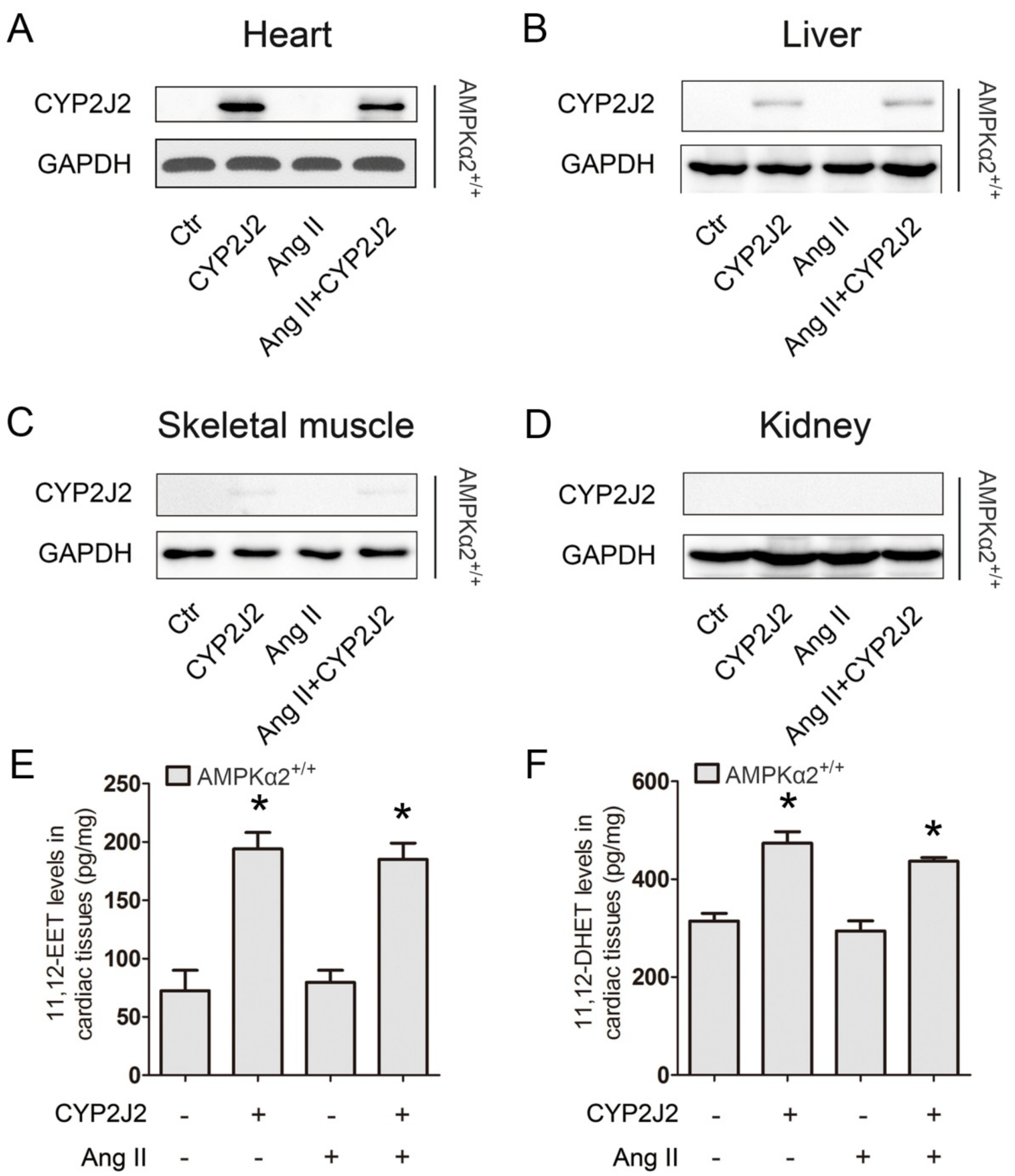

Figure S1. P450 epoxygenase overexpression mediated by rAAV9 and quantitative analysis of 11,12-EET and 11,12-DHET in cardiac tissues. AMPKα2$^{+/+}$ mice were first injected with rAAd2/9-CYP2J2 by caudal vein for 2 weeks, and then exposed to a 14-d continuous infusion of Ang II (1mg•kg^{-1}•d^{-1}). **(A-D)** The western blot analyses for expression of CYP2J2 in **(A)** heart, **(B)** liver, **(C)** skeletal muscle and **(D)** kidney tissues from AMPKα2$^{+/+}$ mice. GAPDH was used as a loading control. **(E-F)** ELISA analysis showed the levels of 11,12-EET and 11, 12-DHET in heart tissues from AMPKα2$^{+/+}$ and AMPKα2$^{-/-}$ mice (n= 4-5 for each group). (*P < 0.05 vs control)
