# Supplementary material for: CYP2J2 and its metabolites (epoxyeicosatrienoic acids) attenuate cardiac hypertrophy by activating AMPKα2 and enhancing nuclear translocation of Akt1
Source: Aging Cell. 2016 Jul 14;15(5):940–52. doi: 10.1111/acel.12507 (PMC5013012; doi:10.1111/acel.12507)
Supplement: Supplementary file 2 — Fig. S2 Cardiomyocyte‐specific overexpression of CYP2J2 maintained cardiac function. [file ACEL-15-940-s002.pdf]

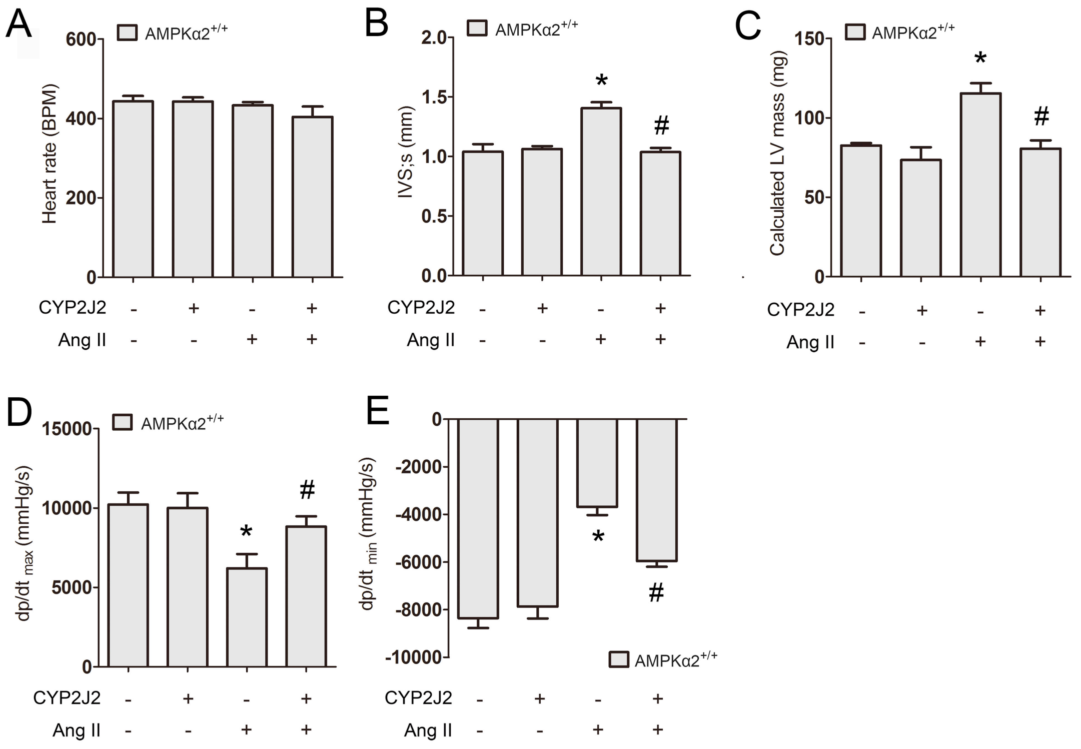

Figure S2. Cardiomyocyte-specific overexpression of CYP2J2 maintained cardiac function. AMPKα2$^{+/+}$ mice were injected in the caudal vein with rAAV9-CYP2J2. After 2 weeks, the mice were exposed to continuous infusion with Ang II or a saline control for 14 days (8-10 mice for each group). Echocardiographic analyses and catheter studies were conducted after infusion with Ang II or saline for 2 weeks. **(A)** Heart rate. **(B)** IVS;s. **(C)** calculated LV mass. **(D)** dP/dt_{max} and **(E)** dP/dt_{min}. The data represent the mean ± SEM from at least four independent experiments (*P < 0.05 vs control group; $^{\#}$P < 0.05 vs Ang II group).
