# Supplementary material for: CYP2J2 and its metabolites (epoxyeicosatrienoic acids) attenuate cardiac hypertrophy by activating AMPKα2 and enhancing nuclear translocation of Akt1
Source: Aging Cell. 2016 Jul 14;15(5):940–52. doi: 10.1111/acel.12507 (PMC5013012; doi:10.1111/acel.12507)
Supplement: Supplementary file 3 — Fig. S3 Different effects of the four regioisomeric forms of EET on PE‐induced cardiac hypertrophy in vitro. [file ACEL-15-940-s003.pdf]

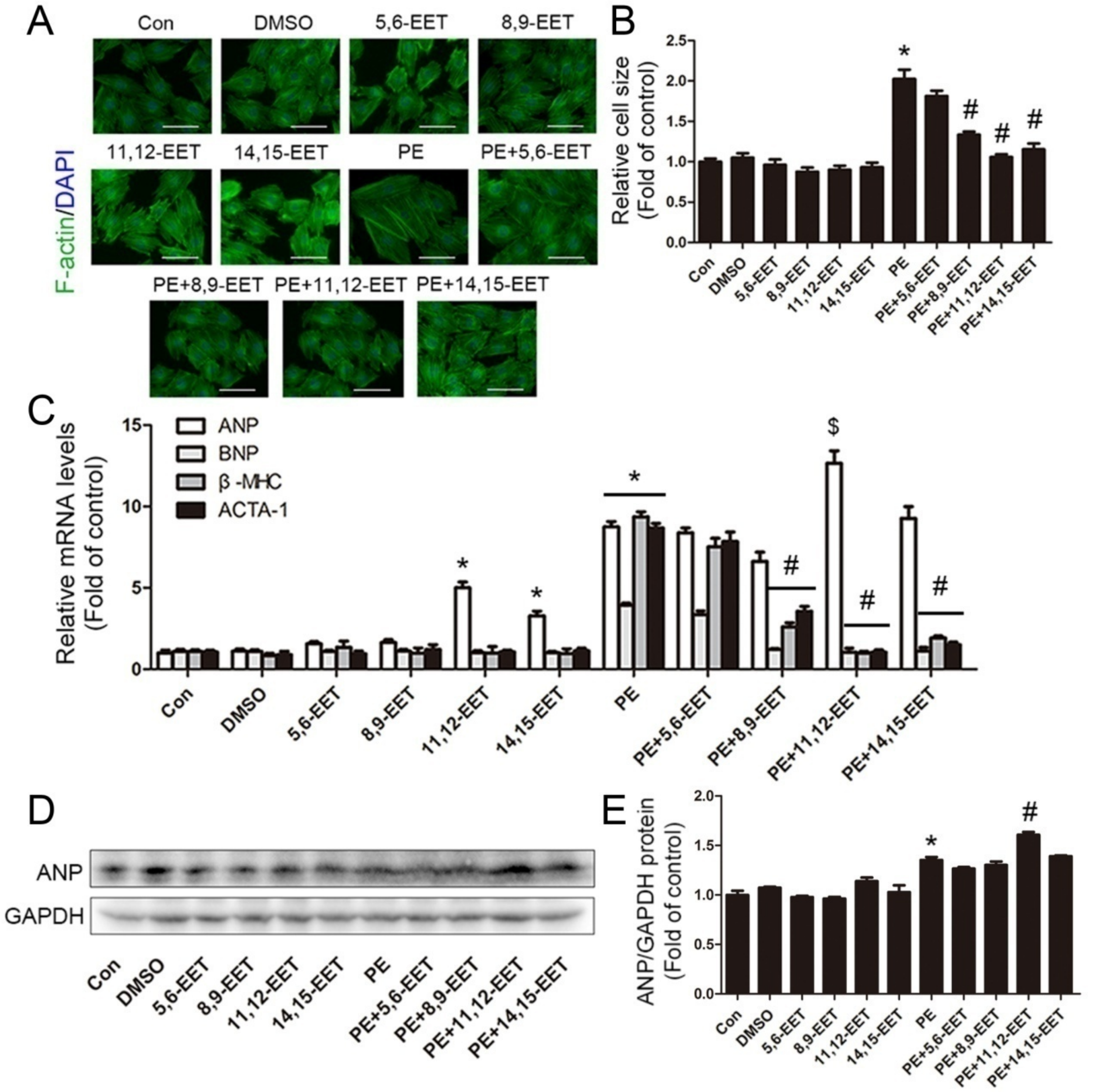

Figure S3. Different effects of the four regioisomeric forms of EET on PE-induced cardiac hypertrophy in vitro. Adult mouse cardiomyocytes were pre-treated with four regioisomeric forms of EET (5,6-EET, 8,9-EET, 11,12-EET and 14,15-EET) (1μmol/L), respectively, and then stimulated with PE (50μmol/L) for 24 h. **(A)** Representative images of cells from different groups immunostained for f-actin (green) and for the nuclear marker DAPI (blue) (Scale bar: 100μm). **(B)** Quantification of the size of cardiomyocytes for each group (30 cells/condition in each preparation; four independent preparations). **(C)** RT-PCR analyses of the relative expression of ANP, BNP, β-MHC and ACTA1 in mouse cardiomyocytes subjected to the indicated treatments. **(D)** Analyses of ANP protein expression by western blotting. GAPDH was used as a loading control. **(E)** The intensity of the western blot signal was quantified

and is shown as relative protein expression after normalization to GAPDH. The data represent the mean ± SEM from at least four independent experiments. (*P < 0.05 vs control; #P < 0.05 vs PE group)
