# Supplementary material for: CYP2J2 and its metabolites (epoxyeicosatrienoic acids) attenuate cardiac hypertrophy by activating AMPKα2 and enhancing nuclear translocation of Akt1
Source: Aging Cell. 2016 Jul 14;15(5):940–52. doi: 10.1111/acel.12507 (PMC5013012; doi:10.1111/acel.12507)
Supplement: Supplementary file 4 — Fig. S4 Overexpression of CYP2J2 mildly attenuated systolic blood pressure and increased ANP expression after Ang II infusion in AMPKα2+/+ mice. [file ACEL-15-940-s004.pdf]

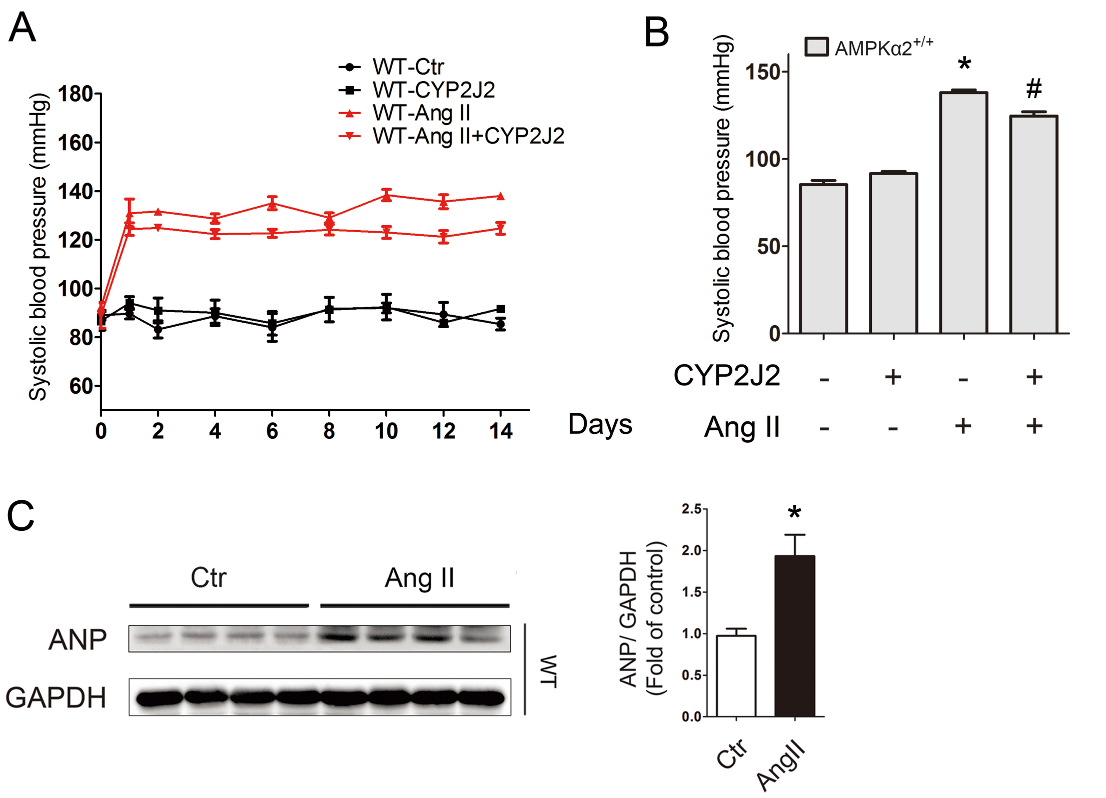

Figure S4. Overexpression of CYP2J2 mildly attenuated systolic blood pressure and increased ANP expression after Ang II infusion in AMPKα2$^{+/+}$ mice. AMPKα2$^{+/+}$ mice were first injected with rAAd9-CYP2J2 by caudal vein for 2 weeks, and then exposed to a 14-d continuous infusion of Ang II (1mg•kg^{-1}•d^{-1}). **(A)** Dynamic systolic blood pressure (SBP) in WT mice was measured by tail-cuff method (n= 4-5 in each group). **(B)** Averaged SBP in each group. **(C) Left,** western blot analyses of protein expression of ANP in control and Ang II groups. GAPDH was used as a loading control. **Right,** the intensity of the Western blot signal was quantified and is shown as relative protein expression after normalization to GAPDH. (*$P < 0.05$ vs control; $^{\#}P < 0.05$ vs Ang II)
