# Supplementary material for: CYP2J2 and its metabolites (epoxyeicosatrienoic acids) attenuate cardiac hypertrophy by activating AMPKα2 and enhancing nuclear translocation of Akt1
Source: Aging Cell. 2016 Jul 14;15(5):940–52. doi: 10.1111/acel.12507 (PMC5013012; doi:10.1111/acel.12507)
Supplement: Supplementary file 5 — Fig. S5 Overexpression of CYP2J2 in cardiomyocytes exerts stronger antihypertrophic effects than administration of hydralazine in AMPKα2+/+ mice. [file ACEL-15-940-s005.pdf]

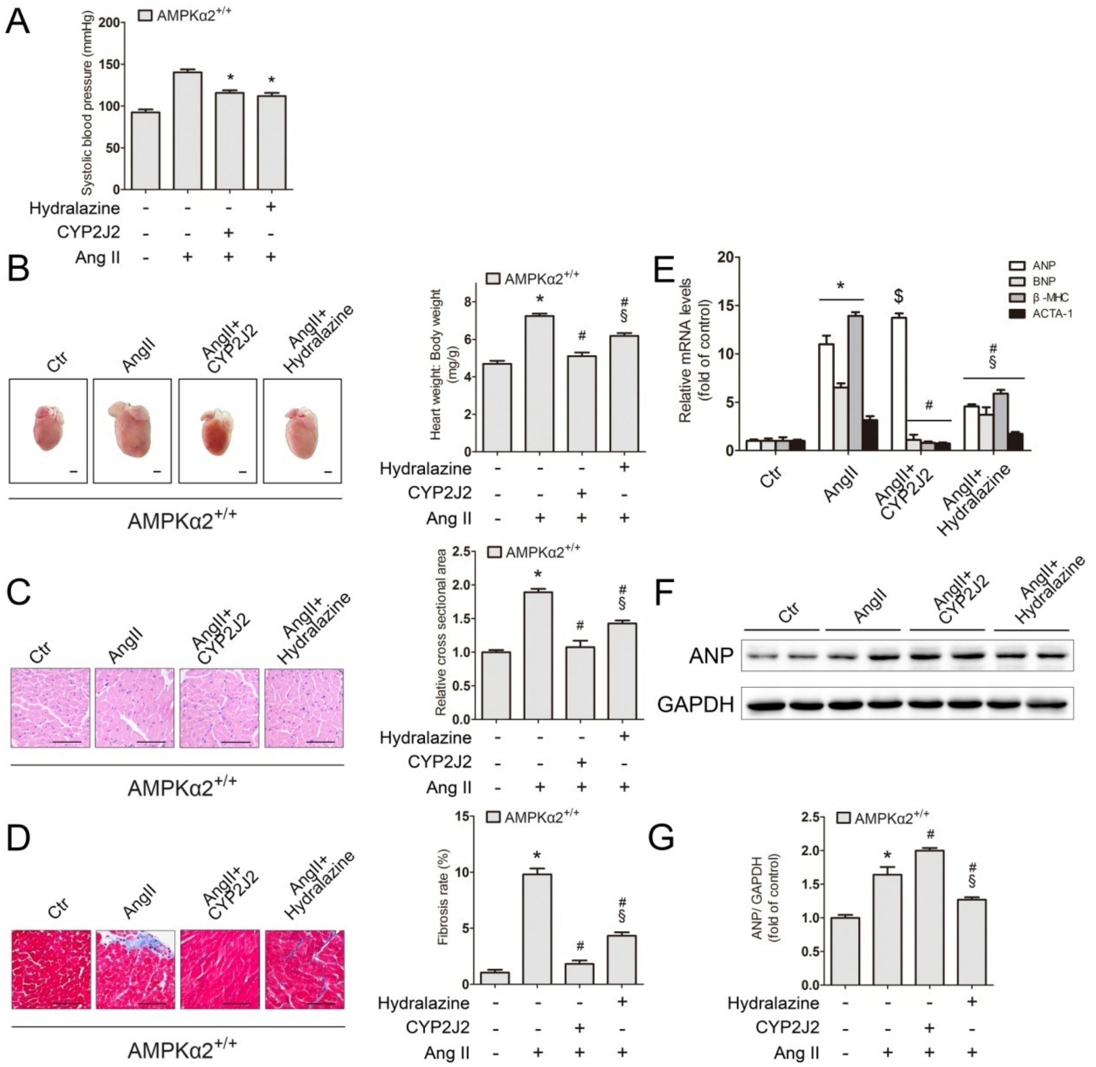

Figure S5. Overexpression of CYP2J2 in cardiomyocytes exerts stronger anti-hypertrophic effects than administration of hydralazine in WT mice. AMPKα2$^{+/+}$ mice were first injected with rAAd9-CYP2J2 by caudal vein for 2 weeks, and then exposed to a 14-d continuous infusion of Ang II (1mg•kg^{-1}•d^{-1}). Additionally, a part of mice with infusion of AngII were treated with hydralazine in drinking water (100mg/L) (n=5 in each group). **(A)** Systolic blood pressure (SBP) in AMPKα2$^{+/+}$ mice was measured every 2 days by tail-cuff method (n= 5 in each group). And averaged SBP in each group was shown. **(B) Left,** The gross morphology of adult hearts from WT mice 2 weeks after Ang II infusion (Scale bar: 1mm). **Right**, Heart weight: body weight ratios of adult WT mice after infusion with Ang II or saline control for 2 weeks. **(C) Left,** H&E staining of sections of adult hearts from AMPKα2$^{+/+}$ mice after infusion with Ang II or saline control for 2 weeks (Scale bar: 100μm). **Right,** Quantification of the size of

cardiomyocytes by measurement of the cross-sectional area on H&E-stained sections. More than 250 cells from three different hearts were analyzed per group. **(D) Left,** Masson trichrome staining of adult hearts from AMPKα2$^{+/+}$ mice after infusion with Ang II or saline control for 2 weeks. The blue area indicates collagen fibers (Scale bar: 100μm). **Right,** Quantification of the rate of cardiac fibrosis by measurement of the area of collagen deposition. **(E)** RT-PCR analyses of relative expression of ANP, BNP, β-MHC and ACTA1 genes from the hearts of mice exposed to the indicated conditions. **(F)** Western blotting analyses showing the expression of the ANP protein in each group. GAPDH was used as a loading control. **(G)** The intensity of the western blot signal was quantified and is shown as relative protein expression after normalization to GAPDH. The data represent the mean ± SEM from at least four independent experiments. (*$P < 0.05$ vs control group; $^{\#}P < 0.05$ vs Ang II group; and $^{\S}P < 0.05$ vs Ang II+CYP2J2 group of mice)
