# Supplementary material for: CYP2J2 and its metabolites (epoxyeicosatrienoic acids) attenuate cardiac hypertrophy by activating AMPKα2 and enhancing nuclear translocation of Akt1
Source: Aging Cell. 2016 Jul 14;15(5):940–52. doi: 10.1111/acel.12507 (PMC5013012; doi:10.1111/acel.12507)
Supplement: Supplementary file 6 — Fig. S6 Overexpression of CYP2J2 in cardiomyocytes increased the activity of AMPK compared with AngII in AMPKα2+/+ mice. [file ACEL-15-940-s006.pdf]

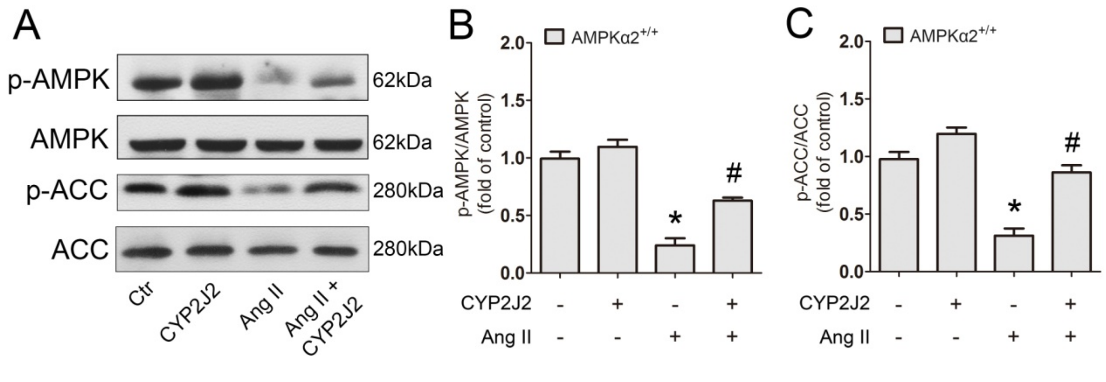

Figure S6. Overexpression of CYP2J2 in cardiomyocytes increased the activity of AMPK compared with Ang II in AMPKα2$^{+/+}$ mice. AMPKα2$^{+/+}$ mice were first injected with rAAd9-CYP2J2 by caudal vein for 2 weeks, and then exposed to a 14-d continuous infusion of Ang II (1mg•kg^{-1}•d^{-1}). **(A)** Western blotting analyses showing the expression of the p-AMPK, AMPK, p-ACC, ACC proteins in the indicated groups. **(B)** The ratio of the p-AMPK/AMPK was quantified. **(C)** The ratio of the p-ACC/ACC was quantified. (*P < 0.05 vs control group; $^{\#}$P < 0.05 vs Ang II group)
