# Supplementary material for: CYP2J2 and its metabolites (epoxyeicosatrienoic acids) attenuate cardiac hypertrophy by activating AMPKα2 and enhancing nuclear translocation of Akt1
Source: Aging Cell. 2016 Jul 14;15(5):940–52. doi: 10.1111/acel.12507 (PMC5013012; doi:10.1111/acel.12507)
Supplement: Supplementary file 7 — Fig. S7 Cytoplasmic and nuclear Akt1 expression in AMPKα2+/+ and AMPKα2−/− heart extracts in basal state and after Ang II treatment. [file ACEL-15-940-s007.pdf]

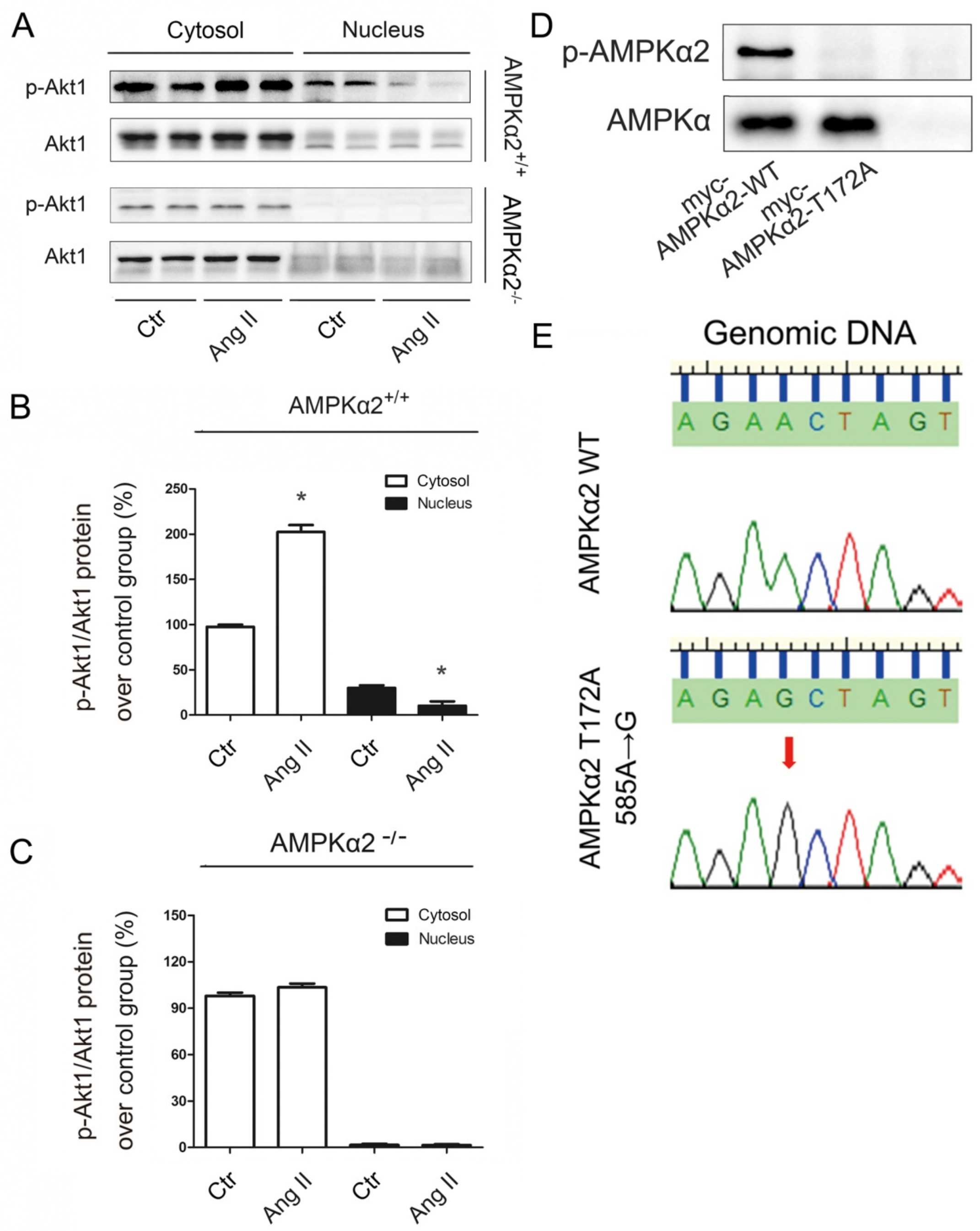

Figure S7. Cytoplasmic and nuclear Akt1 expression in AMPKα2$^{+/+}$ and AMPKα2$^{-/-}$ heart extracts in basal state and after Ang II treatment. AMPKα2$^{-/-}$ and littermate AMPKα2$^{+/+}$ mice were first injected with rAAd9-CYP2J2 by caudal vein for 2 weeks, and then exposed to a 14-d continuous infusion of Ang II (1mg•kg^{-1}•d^{-1}). **(A)** Representative western blot to determine the cytoplasmic and nuclear expression levels of p-Akt1 and Akt1 in AMPKα2$^{+/+}$ and AMPKα2$^{-/-}$ heart extracts in basal state and after Ang II treatment. **(B)** The densitometry of a total of four samples for p-Akt1/Akt1 in each group in AMPKα2$^{+/+}$ mice is depicted. **(C)** The densitometry of a

total of five samples for p-Akt1/Akt1 in each group in AMPKα2$^{-/-}$ mice depicted. **(D)** Myc-AMPKα2-WT or myc-AMPKα2-T172A plasmid was transfected in HEK293T cell by Lipo2000, respectively. Western blotting showed the identification of myc-AMPKα2-WT and myc-AMPKα2-T172A plasmid. **(E)** Identification of the myc-AMPKα2-WT and mutant myc-AMPKα2-T172A plasmid by sequencing. All data represent the mean ± SEM from at least four independent experiments. (*P < 0.05 vs control; $^{\#}$P < 0.05 vsAng II; †P < 0.05 vs Ang II+CYP2J2 group in AMPKα2$^{+/+}$ mice)
