# Supplementary material for: CYP2J2 and its metabolites (epoxyeicosatrienoic acids) attenuate cardiac hypertrophy by activating AMPKα2 and enhancing nuclear translocation of Akt1
Source: Aging Cell. 2016 Jul 14;15(5):940–52. doi: 10.1111/acel.12507 (PMC5013012; doi:10.1111/acel.12507)
Supplement: Supplementary file 8 — Fig. S8 Proposed model for the signaling pathway by which CYP2J2 or 11,12‐EET attenuates cardiac hypertrophic response. [file ACEL-15-940-s008.pdf]

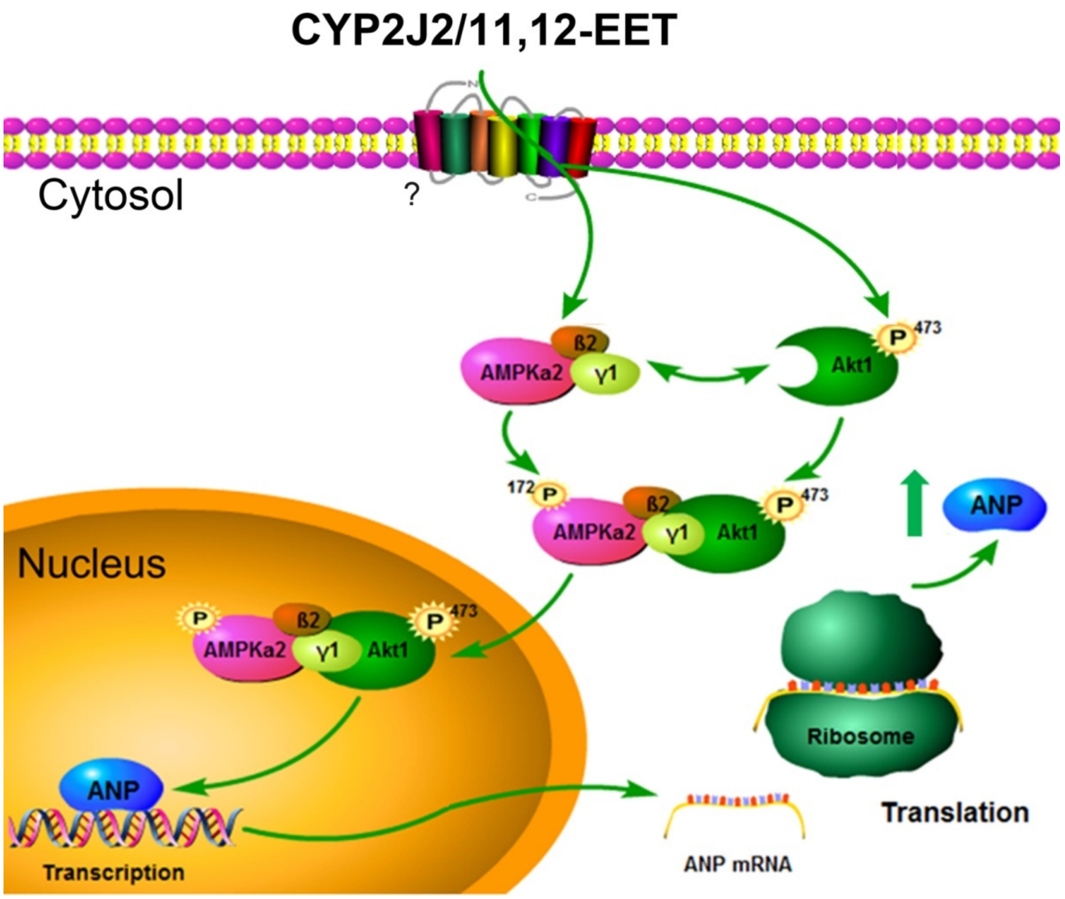

Figure S8. Proposed model for the signaling pathway by which CYP2J2 or 11,12-EET attenuates cardiac hypertrophic response. Overexpression of CYP2J2 or long-term stimulation with 11, 12-EET induces an activation of AMPKα2 in response for cardiac hypertrophy. CYP2J2 or 11, 12-EET first activates AMPKα2 containing β2 and γ1 subunits and activated AMPKα2 containing β2 and γ1 rapidly binds with Akt1 through the direct binding of γ1 subunit and Akt1 protein kinase domain, leading to translocation of p-Akt1 to the nucleus. Nuclear p-Akt1 re-programmed the gene encoded ANP to play its role against cardiac hypertrophy.
