# Supplementary material for: CYP2J2 and its metabolites (epoxyeicosatrienoic acids) attenuate cardiac hypertrophy by activating AMPKα2 and enhancing nuclear translocation of Akt1
Source: Aging Cell. 2016 Jul 14;15(5):940–52. doi: 10.1111/acel.12507 (PMC5013012; doi:10.1111/acel.12507)
Supplement: Supplementary file 9 — Table S1 Primers for quantitative real‐time PCR. [file ACEL-15-940-s009.doc]

| **Table S1** Primers for quantitative real-time PCR | | | |
| --- | --- | --- | --- |
|
| Gene name | Gene ID | Forward or 5' primer | Reverse or 3' primer |
| mouse ANP (*Nppa*) | NM_008725.2 | AGTGCGGTGTCCAACACAGAT | TCCTTGGCTGTTATCTTCGGTA |
| mouse BNP (*Nppb*) | NM_008726.4 | CCTAGCCAGTCTCCAGAGCAAT | CTTCCTACAACAACTTCAGTGCGT |
| mouse β-MHC (*Myh7*) | NM_080728.2 | CTACAGGCCTGGGCTTACCT | TCTCCTTCTCAGACTTCCGC |
| mouse ACTA-1 (*Acta1*) | NM_001272041.1 | CTAGACACACTCCACCTCCA | GTCAGTTTACGATGGCAGCA |
| mouse GAPDH (*Gapdh*) | NM_008084.2 | CAAAATGGTGAAGGTCGGTGTG | TGATGTTAGTGGGGTCTCGCTC |
| Rat ANP (*Nppa*) | NM_012612.2 | CTTCGGGGGTAGGATTGAC | CTTGGGATCTTTTGCGATCT |
| Rat BNP (*Nppb*) | NM_031545.1 | CAGAAGCTGCTGGAGCTGATAAG | TGTAGGGCCTTGGTCCTTTG |
| Rat β-MHC (*Myh7*) | NM_017240.1 | ATCAAGGGAAAGCAGGAAGC | CCTTGTCTACAGGTGCATCA |
| Rat ACTA-1 (*Acta1*) | NM_019212.2 | AGGACCTGTACGCCAACAAC | GCCAGAGCTGTGATCTCCTT |
| Rat GAPDH (*Gapdh*) | NM_017008.3 | GGCAAGTTCAACGGCACAG | CGCCAGTAGACTCCACGACAT |
