# Supplementary material for: CYP2J2 and its metabolites (epoxyeicosatrienoic acids) attenuate cardiac hypertrophy by activating AMPKα2 and enhancing nuclear translocation of Akt1
Source: Aging Cell. 2016 Jul 14;15(5):940–52. doi: 10.1111/acel.12507 (PMC5013012; doi:10.1111/acel.12507)
Supplement: Supplementary file 10 — Table S2 Hemodynamic characteristics of animal groups. [file ACEL-15-940-s010.doc]

| **Table S2** Haemodynamic characteristics of animal groups | | | | | | | | |
| --- | --- | --- | --- | --- | --- | --- | --- | --- |
|
|  | WT-Ctr | WT-AngII | WT-AngII+CYP2J2 | WT-AngII +hydralazine | KO-Ctr | KO-AngII | KO-AngII+CYP2J2 | KO-AngII +hydralazine |
|
| HR (b.p.m.) | 428.5±22.1 | 413±11.2 | 429.2±23.5 | 443.9±33.2 | 426.3±20.0 | 419.6±25.2 | 443.1±30.2 | 447.5±26.5 |
| d*P*/dt_max_ (mmHg/s) | 11397±323.8 | 5022±365.7* | 10355±716.2^#^ | 7758±233.9^#†^ | 10987±376.5 | 4605±261.7* | 6183±227.1 | 7690±327.8^#†^ |
| d*P*/dt_min_ (mmHg/s) | -8416 ±313.6 | -3682±119.8* | -9748±528.2^#^ | -5193±363.4^†^ | -8669±404.6 | -3897±305.8* | -4408±540.6 | -5346±395.1 |
| *P*_max_ (mmHg) | 102.9±1.6 | 73.7±4.7* | 108.4±3.3^#^ | 98.3±2.5^#^ | 110.6±4.1 | 78.1±5.7* | 76.0±3.7 | 97.4±4.6^#,†^ |
| *P*_es_(mmHg) | 95.5±2.5 | 60.1±2.2* | 84.5±3.5^#^ | 76.4±2.4^#^ | 95.2±3.9 | 57.4±2.6* | 61.5±2.6 | 75±3.0^#,†^ |
| Values represent mean ± SEM; n = 5 for each group. | | | | | | | | |
| HR, heart rate; d*P*/dt_max_, peak instantaneous rate of leftventricular pressure increase; d*P*/dt_min_, peak instantaneous rate of leftventricular pressure increase decline; | | | | | | | | |
| *P*_max_,peak systolic pressure; *P*_es_, end systolic pressure. | | | | | | | | |
| *P< 0.05 vs. corresponding Control group | | | | | | | | |
| ^#^P< 0.05 vs. corresponding AngII group | | | | | | | | |
| ^†^P< 0.05 vs. corresponding (AngII + CYP2J2) group | | | | | | | | |
