# Supplementary material for: CYP2J2 and its metabolites (epoxyeicosatrienoic acids) attenuate cardiac hypertrophy by activating AMPKα2 and enhancing nuclear translocation of Akt1
Source: Aging Cell. 2016 Jul 14;15(5):940–52. doi: 10.1111/acel.12507 (PMC5013012; doi:10.1111/acel.12507)
Supplement: Supplementary file 11 — Table S3 Echocardiographic characteristics of animal groups. [file ACEL-15-940-s011.doc]

| **Table S3** Echocardiographic characteristics of animal groups | | | | | | | | |
| --- | --- | --- | --- | --- | --- | --- | --- | --- |
|  |  |  |  |  |  |  |  |  |
|  | WT-GFP | WT-CYP2J2 | WT-Ang II | WT-AngII +CYP2J2 | KO-GFP | KO-CYP2J2 | KO-Ang II | KO-AngII+ |
|  |  |  |  |  |  |  |  | CYP2J2 |
| HR (b.p.m.) | 443.0±13.65 | 442.5 ± 10.57 | 433.0 ±8.09 | 403.8 ±26.28 | 477.8±37.58 | 441.8 ±25.36 | 442.7±14.81 | 456.3 ±20.70 |
| IVS;d (mm) | 0.81±0.04 | 0.80 ±0.04 | 1.02 ±0.05* | 0.76 ±0.07^#^ | 0.78 ±0.07 | 0.76 ±0.03 | 1.07 ±0.04* | 1.03 ±0.05^†^ |
| LVID;d (mm) | 3.60 ±0.14 | 3.59 ±0.13 | 4.23 ±0.08* | 4.08 ±0.11 | 3.83 ±0.08 | 3.60 ±0.14 | 4.37 ±0.11* | 4.33 ±0.15 |
| LVPW;d (mm) | 0.58 ±0.04 | 0.56 ±0.03 | 0.76 ±0.03* | 0.60 ±0.02^#^ | 0.56 ±0.05 | 0.58 ±0.03 | 0.82 ±0.04* | 0.76 ±0.05^†^ |
| IVS;s (mm) | 1.04 ±0.06 | 1.06 ±0.02 | 1.40 ±0.05* | 1.03 ±0.03^#^ | 1.04 ±0.01 | 1.05 ±0.06 | 1.53 ±0.04* | 1.39 ±0.04^†^ |
| LVID;s (mm) | 2.26 ±0.10 | 2.32 ±0.19 | 3.26 ±0.12* | 2.65 ±0.05^#^ | 2.75 ±0.06 | 2.63 ±0.23 | 3.34 ±0.13* | 3.11 ±0.18^†^ |
| LVPW;s (mm) | 0.95 ±0.02 | 0.97 ±0.04 | 1.25 ±0.02* | 0.92 ±0.07^#^ | 0.97 ±0.06 | 0.88 ±0.07 | 1.364±0.05* | 1.29 ±0.06^†^ |
| LV Mass Corrected (mg) | 82.57 ±1.57 | 73.43 ±8.09 | 115.4 ±6.34* | 80.65 ±5.13^#^ | 85.05 ±7.48 | 65.4 ±4.58 | 121.7 ±5.11* | 113.3 ±7.38^†^ |
| %EF | 69.59 ±1.11 | 68.62 ±2.02 | 43.03 ±3.45* | 61.59 ±1.40^#^ | 64.58 ±1.98 | 66.33 ±2.81 | 45.85 ±2.31* | 48.40 ±3.74^†^ |
| % FS | 36.97 ±1.70 | 36.24 ±2.56 | 21.15 ±1.16* | 30.13 ±0.92^#^ | 35.41 ±2.58 | 38.49 ±2.08 | 19.87 ±0.87* | 19.78 ±1.13^†^ |
| Values represent mean+SEM; n ≥ 8 per group. HR,heart rate; IVS;d, interventricular septum at diastole; LVID;d LV internal diameter at diastole; LVPW,d, LV posterior wall thickness at diastole; IVS;s, interventricular septum at systole; LVID;s LV internal diameter at systole; LVPW;s, LV posterior wall thickness at systole; LV Mass Corrected, corrected left ventricular mass; EF, ejection fraction; FS, fractional shortening. *P <0.05 GFP group; ^#^P <0.05 WT-Ang II group; ^†^P <0.05 WT-Ang II+CYP2J2 group | | | | | | | | |
