# Supplementary material for: CYP2J2 and its metabolites (epoxyeicosatrienoic acids) attenuate cardiac hypertrophy by activating AMPKα2 and enhancing nuclear translocation of Akt1
Source: Aging Cell. 2016 Jul 14;15(5):940–52. doi: 10.1111/acel.12507 (PMC5013012; doi:10.1111/acel.12507)
Supplement: Supplementary file 12 — Appendix S1 Extended Experimental Procedures. [file ACEL-15-940-s012.doc]

**Extended Experimental procedures:**

**Echocardiographic analysis**

Mice were anesthetized using a mixture of 1.5% isoflurane and O_2_ (1 to 2 L). Echocardiography was performed as described previously (Yamamo*to et a*l. 2003) with a 13-MHz linear ultrasound transducer. After 2-dimensional left ventricular (LV) short-axis images were obtained, M-mode traces were acquired for measurements of diastolic and systolic LV wall thickness and chamber dimensions, echocardiography-derived LV masses, ejection fraction, fractional shortening, and heart rates. Measurements were averaged from 5 consecutive cardiac cycles.

**Hemodynamic measurements of left ventricular (LV) function**

Measurements of LV function were performed using Millar catheter system as described before (Shiou*ra et a*l. 2007).

**Antibodies and reagents**

The following commercial antibodies were used: AMPKα1(#2795, 1:1,000 for western blot), AMPKα2(#2532, 1:1,000 for western blot), AMPKα(#2535, 1:1,000 for western blot), AMPKβ1 (#12063, 1:1,000 for western blot), AMPKβ2 (#4148, 1:1,000 for western blot), AMPKγ1 (#4187, 1:1,000 for western blot), AMPKγ2 (#2536, 1:1,000 for western blot), AMPKγ3 (#2550, 1:1,000 for western blot), phospho-Akt1 (Ser 473)(#12694, 1:1,000 for western blot), Akt1 (#2967, 1:1,000 for western blot), phospho-Akt2 (Ser 474)(#8599, 1:1,000 for western blot), Akt2 (#2962, 1:1,000 for western blot), phospho-Akt3 (Ser 472)(#9611, 1:1,000 for western blot), Akt3 (#4059, 1:1,000 for western blot), phospho-ACC(#11818, 1:1,000 for western blot), ACC(#3676, 1:1,000 for western blot), GAPDH (#2118, 1:1,000 for western blot, CST, Danvers, MA),phospho-AMPKα2(Thr 172) (ab133448, 1:1,000 for western blot, Abcam, Cambridge, MA), human CYP2J2 (sc-137100, 1:1,000 for western blot), ANP (sc-20158, 1:1,000 for western blot), Lamin B1(sc-20682, 1:1,000 for western blot), myc-tag (conjugated to agarose beads, Santa Cruz, CA) and mouse control IgG (Sigma-Aldrich). EETs (5,6-EET, 8,9-EET, 11,12-EET and 14,15-EET) and EET antagonist 14,15-EEZE were from Cayman Chemical company (Cayman Chemical, Ann Arbor, MI). AMPK activator 5-aminoimidazole-4-carboxamide ribonucleotide (AICAR) and Angiotensin II were from Sigma (Sigma-Aldrich, St. Louis, MO). Akt1 selective inhibitor MK2206 (dihydrochloride) was supplied by Selleck Company (Houston, TX). The mouse plasma ANP level was measured with an enzyme-linked immunosorbent assay (RayBiotech, Inc., Norcross, GA) and 11,12-EET/DHET levels in cardiac tissues were detected by using a commercial Elisa kit (Detroit R&D Inc., Detroit, MI, USA).

**Cell area measurement**

Plated cardiomyocytes (3 × 10^4^ cells per 15.6 mm well of 24 well plates) were treated in accordance with the experimental protocol. The cells were stained with Actin-Tracker Green according to the manufacturer’s instructions (Beyotime, Shanghai, China). At the end of the experiment, the cells were viewed using a Leica DMILLED inverted microscope (Wetzlar, Germany), equipped with a 3.2 MP scientiﬁc grade digital microscopy camera. Six to eight random photographs were taken from each well, and at least, 40 individual cell size measurements were made from each group using Image-Pro Plus 6.0 software (ipp6.0).

**Plasmid Constructs and Transfection.**

Human cDNAs encoding wild type AMPKα2 was purchased from Origene (#RC210226, Origene Tech. Inc, MD). The T172A mutant was created using the Quick Change Site-Directed Mutagenesis Kit (Stratagene, La Jolla, CA) and the following primers: 3' CCTCTAGGCGTTGATCGAGAGTCTTTAAGTGGTA 5' (F) and 5' GGAGATCCGCAACTAGCTCTCAGAAATTCACCAT 3' (R). Flag-AMPKγ1 was purchased fromVigene (#CH842000, ViGene Biosciences Inc, Rockville, MD). Flag-AMPKα2 and Flag-AMPKβ2 were constructed by using PCR amplification inserting AMPKα2 or AMPKβ2 into the pENTER vector. Myc-Akt1-PH, myc-Akt1-PK and myc-Akt1-AGC plasmids were constructed by using PCR amplification from the plasmid expressing myc-tagged full length human Akt1 (#RC220257, Origene Tech. Inc, MD, USA) and the following primers: *1)* Akt1-PH (F): 5' GGC GTG TAC GGT GGG ATG AGC GAC GTG GCT 3' and Akt1-PH (R): 5' TGC TTA TAT AGA CCT CTT GAG GCC GTC AGC 3'; *2)* Akt1-PK (F): 5' GGC GTG TAC GGT GGG TTT GAG TAC CTG AAG 3' and Akt1-PK (R): 5' TGC TTA TAT AGA CCT AAA GAA GCG ATG CTG 3'; *3)* Akt1-AGC (F): 5' GGC GTG TAC GGT GGG TTT GCC GGT ATC GTG TG 3' and Akt1-AGC (R):5' TTA TAT AGA CCT TCA GGC CGT GCC GCT GGC 3'. The plasmid expressing GST-tagged human Akt1 was constructed by using PCR amplification and inserting it into the BamHI/NotI site of pEBG vector.

Transfection was performed with Lipofectamine 2000 reagent (Invitrogen, Carlsbad, CA) according to the manufacturer’s instructions. HEK 293T Cells were plated in 100 mm dish at a density of approximately 1.2 × 10^6^ cells/ dish. The cells were harvested 48h after transfection with myc-AMPK-WT, myc-AMPK-T172A, Flag-AMPKα2, Flag-AMPKβ2, Flag-AMPKγ1, GST-Akt1, myc-Akt1, myc-Akt1-PH, myc-Akt1-PK and myc-Akt1-AGC plasmids. Plasmid DNA for transient transfection was prepared with the TaKaRa Mini BEST Plasmid Purification Kit (TakaraBio Inc., Dalian, China) and AMPKα2 siRNA was chemically synthesized. The mutant AMPK plasmids were generated by site-directed mutagenesis. All mutations were verified by sequencing.

**Nuclear extract and cytosol preparation**

Cardiomyocyte nuclear extracts and cytosol preparation were prepared as previously described (Camper-Kir*by et a*l. 2001).

**Immunohistochemical analysis**

The Akt1 Immunostaining was performed as previously described (*Ng et a*l. 2012). In brief, hearts were arrested in diastole by perfusion with 5M KCL at 100 mmHg. Tissue was fixed in PFA (1%) and underwent normal histological preparation into paraffin blocks and onto slides. 5 µm sections were then deparaffinized and rehydrated through a series of EtOH concentrations. Cardiomyocyte area was quantified in sections stained with H&E. Images were obtained on a Leica laser scanning microscope (Nikon) at 200x magnification. Cryosections were used for immunostaining with antibodies total Akt1 (Cell Signaling Technologies). 4’-6-Diamidino-2-phenylindole (DAPI; Sigma) was used for nuclear staining.

**Co-immunoprecipitation and GST pull-down experiments**

Nuclear or cytosol proteins were extracted from cardiomyocytes or HEK 293T cells. Primary antibody was covalently immobilized on protein A/G agarose using the Pierce Crosslink Immunoprecipitation Kit according to the manufacturer’s instructions (Thermo Fisher Scientific Inc., Waltham, MA). After overnight incubation at 4 °C, 40 µl of protein G-conjugated agarose beads was added and incubated for 2 h at 4 °C. The beads were washed five times with PBS and resuspended in 60 µl of Laemmli buffer. The immunoprecipitated samples were subjected to immunoblotting using specific primary antibodies and a conformation-specific secondary antibody that recognizes only native IgG (Cell Signaling). For purification of recombinant GST fusion proteins, GSH-Sepharose was incubated with lysates and washed as immunoprecipitation. The precipitates were then analyzed by immunoblots.

**Immunoblotting**

Cell Lysates were centrifuged at 13,200 r.p.m. for 15 min at 4 °C. Electrophoresis was performed on Laemmli SDS-polyacrylamide gels. Separated proteins were transferred from the gels to polyvinylidene fluoride membranes by semidry electrotransfer, and immunoblotting was performed as previously described (Shima*no et a*l. 2011).

**Quantification of mRNA by RT-PCR**

Total RNA was extracted from myocardial tissues in mice and neonatal rat cardiomyocytes using Trizol (Life Technologies Inc., Carlsbad, California, USA). Expression levels of mRNA for ANP, BNP, β-MHC and ACTA1 were quantiﬁed by a real-time two-step RT-PCR assay with use of SYBR green chemistry, based on the 5’-nuclease activity of Taq polymerase, and a sequence detection system (GeneAmp7900, HT Applied Bio systems, Foster City, CA). The PCR primers are listed in Table S1. All samples were processed in triplicate reactions and underwent 40 rounds of amplification as follows: 40 cycles of a two-step PCR (95°C for 30 sec, 58°C for 30 sec) after initial denaturation (95°C for 5 min). A melting curve was also performed to check for undesirable amplification. For accurate gene expression measurements, the sequences of primers used for PCR amplication are shown in Table S1. GAPDH was used as reference endogenous genes to normalize the results. Each RT-PCR was performed on at least four different experimental samples.

**Reference**

Camper-Kirby D, Welch S, Walker A, Shiraishi I, Setchell KD, Schaefer E, Kajstura J, Anversa P, Sussman MA (2001). Myocardial Akt activation and gender: increased nuclear activity in females versus males. *Circulation research*. **88**, 1020-1027.

Ng R, Song G, Roll GR, Frandsen NM, Willenbring H (2012). A microRNA-21 surge facilitates rapid cyclin D1 translation and cell cycle progression in mouse liver regeneration. *The Journal of clinical investigation*. **122**, 1097-1108.

Shimano M, Ouchi N, Nakamura K, van Wijk B, Ohashi K, Asaumi Y, Higuchi A, Pimentel DR, Sam F, Murohara T, van den Hoff MJ, Walsh K (2011). Cardiac myocyte follistatin-like 1 functions to attenuate hypertrophy following pressure overload. *Proceedings of the National Academy of Sciences of the United States of America*. **108**, E899-906.

Shioura KM, Geenen DL, Goldspink PH (2007). Assessment of cardiac function with the pressure-volume conductance system following myocardial infarction in mice. *American journal of physiology. Heart and circulatory physiology*. **293**, H2870-2877.

Yamamoto S, Yang G, Zablocki D, Liu J, Hong C, Kim SJ, Soler S, Odashima M, Thaisz J, Yehia G, Molina CA, Yatani A, Vatner DE, Vatner SF, Sadoshima J (2003). Activation of Mst1 causes dilated cardiomyopathy by stimulating apoptosis without compensatory ventricular myocyte hypertrophy. *The Journal of clinical investigation*. **111**, 1463-1474.
